# A comprehensive *in silico* investigation into the pathogenic SNPs in the RTEL1 gene and their biological consequences

**DOI:** 10.1371/journal.pone.0309713

**Published:** 2024-09-06

**Authors:** Rifah Rownak Tanshee, Zimam Mahmud, A. H. M. Nurun Nabi, Mohammad Sayem

**Affiliations:** 1 Department of Mathematics and Natural Sciences, BRAC University, Badda, Dhaka, Bangladesh; 2 Department of Biochemistry and Molecular Biology, University of Dhaka, Dhaka, Bangladesh; The University of Texas Rio Grande Valley, UNITED STATES OF AMERICA

## Abstract

The Regulator of Telomere Helicase 1 (RTEL1) gene encodes a critical DNA helicase intricately involved in the maintenance of telomeric structures and the preservation of genomic stability. Germline mutations in the RTEL1 gene have been clinically associated with Hoyeraal-Hreidarsson syndrome, a more severe version of Dyskeratosis Congenita. Although various research has sought to link RTEL1 mutations to specific disorders, no comprehensive investigation has yet been conducted on missense mutations. In this study, we attempted to investigate the functionally and structurally deleterious coding and non-coding SNPs of the RTEL1 gene using an *in silico* approach. Initially, out of 1392 nsSNPs, 43 nsSNPs were filtered out through ten web-based bioinformatics tools. With subsequent analysis using nine *in silico* tools, these 43 nsSNPs were further shortened to 11 most deleterious nsSNPs. Furthermore, analyses of mutated protein structures, evolutionary conservancy, surface accessibility, domains & PTM sites, cancer susceptibility, and interatomic interaction revealed the detrimental effect of these 11 nsSNPs on RTEL1 protein. An in-depth investigation through molecular docking with the DNA binding sequence demonstrated a striking change in the interaction pattern for F15L, M25V, and G706R mutant proteins, suggesting the more severe consequences of these mutations on protein structure and functionality. Among the non-coding variants, two had the highest likelihood of being regulatory variants, whereas one variant was predicted to affect the target region of a miRNA. Thus, this study lays the groundwork for extensive analysis of RTEL1 gene variants in the future, along with the advancement of precision medicine and other treatment modalities.

## Introduction

Regulator of telomere elongation helicase 1 (RTEL1) is an essential iron-sulfur (FeS)-containing DNA helicase, which is a member of the DEAH subfamily of the Superfamily 2 (SF2) helicases and also categorized as a RAD3-like helicase with a 5′ to 3′ helicase activity [1]. It is located at chromosome 20q13.33 and contains thirty-five exons. Various isoforms are produced through alternative splicing results in multiple transcript variants, and in humans, the two main isoforms are- isoform 2 (1219 amino acid) and isoform 6 (1300 amino acid); both differ in the C terminal region [2]. RTEL1 is a multidomain protein that includes a RAD3-like helicase domain-containing helicase type 2 ATP binding domain and C terminus (Dead 2 and Helicase C2) domains, DEAH box, PCNA interacting motifs or PIP boxes, Harmonin N-like domains and RING-finger domain [3, 4]. This gene is essential for telomere regulation, DNA repair, and genome stability that interacts with proteins in the shelterin complex to preserve the telomere.

DNA secondary structures such as trinucleotide repeats, G-quadruplexes or the intermediates formed during the 3R process must be processed correctly to maintain genome stability and reduce pathological consequences [4]. Several studies have suggested the role of RTEL1 as an anti-recombinase that combats harmful recombination and limits the crossover in meiosis. The RTEL1 gene maintains the crossover homeostasis by physically separating strand invasion events, which encourages non-crossover repair through synthesis-dependent strand annealing (SDSA). During DNA repair and meiotic recombination procedures, it facilitates the breakdown of D-loop recombination intermediates [1, 5]. Additionally, through resolving G-quadruplexes created during telomere replication, mouse RTEL1 has also been linked to disassembling T loops and preventing telomere fragility, which collectively maintains the dynamics and integrity of the telomere [6]. Besides, one study demonstrated the association of RTEL1 in unwinding trinucleotide repeat to prevent triplet repeat mediated chromosome fragility [7].

R-loops, a co-occurrence known for its intimate relationship between G4-DNA and RNA structures, increase due to deficient functionality of RTEL1 in cells. Several studies have demonstrated that the regulation of G4-DNA/R-loops is facilitated by RTEL1 and cells with depleted RTEL1, observed to have the inability to unwind G4-DNAs, leading to an increase in R-loops formation, which in turn increases the transcription-replication collisions [8]. This may ultimately lead to genome instability and the emergence of cancer.

DNA replication stress, produced by oncogene activation during tumorigenesis, causes G4/R-loop forming loci, for example, common fragile sites (CFSs) and telomeres, to remain under-replicated during interphase, which is compensated through mitotic DNA synthesis (MiDAS) [9]. The mechanism of MiDAS depends on the RTEL1 protein, where the recruitment of RTEL1 to the affected loci is facilitated through SLX4, which in turn assists in attracting RAD52 and POLD3 protein-both essential for MiDAS [9]. This suggests the necessity of RTEL1 in maintaining genomic stability through resolving conflicts between the replication and transcription machinery. On the other hand, the SLX4-RTEL1 complex increases the recruitment of proteins to nascent DNA, strongly associated with active RNA pol II, which also facilitates the co-localization of FANCD2/RNA pol II [10]. Therefore, the interaction of SLX4 and RTEL1 is necessary for replication fork development. This interaction has been observed to be abolished in patients with HHS and cancer [10].

The expression of the RTEL1 gene is found in the testis, appendix, spleen, endometrium, adrenal, prostate, bone marrow, and 20 other tissues. The mutation in the RTEL1 gene has been linked to a variety of human diseases, including dyskeratosis congenita (DC), Hoyeraal-Hreidarsson syndrome, glioma (HHS), glioblastoma, pulmonary fibrosis, bone marrow failure, breast cancer, and other malignancies [4]. The mutation in the RTEL1 gene can cause multiple discrepancies in telomere biology, cellular replication, and DNA repair mechanism. Multiple clinical studies have observed a broader spectrum of clinical complications in patients with DC and HHS who have inherited RTEL1 mutation [2, 11–15]. In addition, the effect of a mutated RTEL1 gene may vary depending on the cell type and the mutation that occurred in the gene [13]. The risk of tumorigenesis or cancer predisposition due to RTEL1 mutations is not only observed in the case of HHS or DC, but interestingly, it has also been connected to the predisposition for brain malignancies like gliomas, astrocytomas, and glioblastomas [16–18]. The RTEL1 gene has thus been suggested to be a tumor suppressor gene for the emergence of brain malignancies [19]. However, recent studies have also shown that the RTEL1 gene locus is amplified in a number of malignancies, including gastrointestinal and breast tumors [20, 21]. In many cellular circumstances, it is conceivable that either overexpression or downregulation of the RTEL1 gene could lead to the formation of cancer or tumorigenesis in many different ways [22–24].

Single nucleotide polymorphism (SNP), a single base substitution in alleles, is the most prevalent type of mutation in the human genome. SNPs occur in approximately every 1,000 base pairs in the genome [25] and can be found in coding and non-coding regions. Variants in the non-coding region have been demonstrated to impact the function of cis or trans-regulatory elements, UTRs, and introns, which might disrupt the affinity of transcription factors, various epigenetic factors, alternative splicing, and mRNA stability [26]. The SNPs in the coding region, particularly missense or non-synonymous SNPs (nsSNPs), have long been a great concern. They result in amino acid substitutions in the protein sequence, thus altering the activity of the protein. According to earlier research, nsSNPs account for about 50% of the mutations linked to a number of genetic illnesses [27, 28], as well as several autoimmune and inflammatory conditions [29–31].

Functional variations caused by SNPs might have deleterious or neutral effects on protein function, with detrimental impacts involving damage to protein structures and gene regulation [32, 33]. Additionally, changes in the protein sequence may ultimately lead to changes in the dynamics, translation, hydrophobicity, charge, shape, and inter/intra protein interactions, endangering cells [34–36]. This information supports the notion that nsSNPs, particularly missense SNPs, are connected to several human disorders [37, 38]. The use of computational methods in recent studies on nsSNPs successfully revealed the possible relevance of mutation in comprehending the molecular pathways of numerous diseases [39–41]. Although the accuracy of these tools is sometimes uncertain, the combined utilization of different algorithms has enabled us to predict the impact of specific mutations reliably [42, 43]. Moreover, computational analysis is essential for primary filtration, as working with a large amount of SNP data in laboratory experiments would be expensive and time-consuming.

Even though the RTEL1 gene has been the subject of multiple genome-wide association studies, most RTEL1 SNPs have not yet been thoroughly studied for their potential to cause disease. It is still unclear how nsSNPs and non-coding SNPs affect the RTEL1 protein in terms of disease etiology. So far, no comprehensive *in silico* analysis of the RTEL1 gene has been conducted to detect SNPs linked to functional and structural changes in the protein. Therefore, in this study, we aim to elucidate the impact of the most deleterious genetic variations of the RTEL1 gene on the protein’s structure and stability and attain molecular-level insights into SNP-mediated protein’s functional divergence.

## Materials and methods

### Data retrieval

The SNP data of RTEL 1 gene was acquired from the available human GRCh37 genome SNPs in NCBI dbSNP [https://www.ncbi.nlm.nih.gov/snp/?term=] database [44], ClinVar [https://www.ncbi.nlm.nih.gov/clinvar/] database [45] and the DisGeNET [https://www.disgenet.org/] database [46]. Relative data about the RTEL1 gene and the amino acid sequence (FASTA format) of RTEL1 protein were collected from NCBI [https://www.ncbi.nlm.nih.gov/] and UniprotKB (Universal Protein Knowledgebase) [https://www.uniprot.org/] databases (UniprotKB-Q9NZ71), respectively [47]. For the analysis of non-coding SNPs, the dataset was collected from Ensembl [https://asia.ensembl.org/index.html] database [48].

### Retrieval of 3D structure and quality checking

The AlphaFold structure of the human RTEL1 protein was retrieved from the UniprotKB (Universal Protein Knowledgebase) [https://www.uniprot.org/] database. The validation of the retrieved structure was checked using the SAVES [https://saves.mbi.ucla.edu/] server. The results of ERRAT, VERIFY, and PROCHECK Ramachandran plot were analyzed to estimate the validation of the AlphaFold structure of the native protein.

### Functional impact prediction

To determine the functional consequences of nsSNPs that were retrieved from the dbSNP database, ten bioinformatics-based web tools, i.e., PMut, SuSPect, PredictSNP, PredictSNP2, SIFT, SNAP2, SNP & GO, PROVEAN, Polyphen2, PANTHER were used to ensure the veracity and stringency of the results. SNPs commonly identified as deleterious by all these ten algorithms were considered high-risk nsSNPs.

PMut [http://mmb.irbbarcelona.org/PMut/] anticipates the pathological mutations on protein sequences, where a score of >0.5 indicates the disease effects of nsSNPs and <0.5 indicates the neutral effects of nsSNPs on the given protein’s functionality [49]. SuSPect [http://www.sbg.bio.ic.ac.uk/~suspect/] (Disease-Susceptibility-based SAV Phenotype Prediction) webserver predicts single amino acid variants associated with the disease with 82% accuracy [50]. PredictSNP [https://loschmidt.chemi.muni.cz/predictsnp/] is a consensus classifier with eight integrated established prediction tools to predict the mutations related to the disease [51]. PredictSNP2 [https://loschmidt.chemi.muni.cz/predictsnp2/] is a unified web platform with six integrated prediction tools that predict SNPs’ pathogenic effect in distinct genomic regions [52]. PredictSNP2 expands on PredictSNP by evaluating the impacts of nucleotide variants across any genomic region, while PredictSNP is limited to analyzing substitutions within amino acid sequences [52]. SIFT (Sorting Intolerant from Tolerant) [https://sift.bii.a-star.edu.sg/] predicts the impact of an amino acid alteration on protein depending on the sequence homology and physical property of amino acids, where score ≤0.05 indicates damaging and >0.05 is tolerant [53]. Next, PROVEAN (Protein Variation Effect Analyzer) [https://www.jcvi.org/research/provean] was used for the prediction of the damaging impact of nsSNPs on protein sequence [54]. The PROVEAN score, which is generated by averaging the delta alignment scores of variants and reference protein query sequence concerning homology sequence, helps to separate the nsSNPs as deleterious (score ≤ -2.5) and neutral (score >-2.5) variants. SNAP2 [https://rostlab.org/services/snap/] is another neutral network-based web tool that gives prediction scores between -100 and +100, which indicates strong neutral to strong impactful variants [55]. SNP & GO (SNP & Gene Ontology) [https://snps.biofold.org/snps-and-go/snps-and-go.html] is an SVM-based classifier that classifies polymorphisms as a neutral variation or disease-associated variation (when probability score >0.5) [56]. Polyphen2 (Polymorphism phenotype v2) [http://genetics.bwh.harvard.edu/pph2/] analyzes the potential effect of amino acid substitution on the function and structure of protein and based on the probabilistic score it provides the result as benign, possibly damaging and probably damaging [57]. PANTHER (Protein Analysis Through Evolutionary Relationship) [http://www.pantherdb.org/tools/csnpScoreForm.jsp] employs the PANTHER-PSEP (Position Specific Evolutionary Preservation) method to distinguish disease-related variants from neutral variants in the human protein. It estimates the likelihood of nsSNPs disrupting protein functionality by calculating the evolutionary preservation of the amino acid residues, where a long preservation period indicates greater chances of nsSNPs causing a functional impact on the protein [58].

### Structural impact prediction

The structural impact of nsSNPs on the RTEL1 protein was analyzed using nine web tools. Per two distinct categories of tools, the prediction approach was split into two parts. One category of tools was selected for predicting the change in stability, where seven different tools, including DUET, mCSM, SDM, I-Mutant, INPS-MD, MUpro, and Dynamut2 were employed. On the other hand, the following group of tools relied on the prediction of phenotypic effects using two separate web servers, including HOPE and MudPred2.

DUET [http://biosig.unimelb.edu.au/duet/] predicts the alteration in the stability of protein due to the introduced mutation by combining the SDM and mCSM approaches; therefore, both SDM and mCSM predicted results come together with DUET [59]. In this tool, the server gives the result of the change in folding free energy or value of ΔΔG in kcal/mol by subtracting ΔG mutant from ΔG wild type where the negative value indicates destabilization, and a positive value indicates stabilization of the structure. MuPro [http://mupro.proteomics.ics.uci.edu/] predicts the effects of a single-site amino acid substitution on the stability of protein with 84% accuracy using protein sequence and mutation information [60]. I-Mutant 2.0 [https://folding.biofold.org/i-mutant/i-mutant2.0.html] assesses the protein stability change from a given protein sequence and provides information about the state of stability as a decrease or increase in stability upon possible mutation along with Reliability Index [61]. The INPS-MD (Impact of Non-synonymous mutations on Protein Stability-Multi Dimension) [https://inpsmd.biocomp.unibo.it/inpsSuite/default/index] can also predict the stability change of protein from both protein sequence and structure [62]. The stability change of the protein was further analyzed through Dynamut2 [https://biosig.lab.uq.edu.au/dynamut2/] prediction submission panel. Dynamut2 predicts the likely effects of an amino acid alteration on the stability of a protein by employing normal mode analysis and graph-based models to take snapshots of molecular movements in cellular conditions [63].

The web server MutPred2 [http://mutpred2.mutdb.org/] uses machine learning-based algorithms that enable the prediction of pathogenicity of amino acid substitutions in proteins with a probabilistic score along with a list of specific alterations of the molecular mechanism [64]. The effects of harmful nsSNPs on protein structure were examined using the HOPE [http://www.cmbi.ru.nl/hope/home] server. By combining data from numerous sources, such as sequence annotations, tertiary structure, homology models from the Distributed Annotation System (DAS) servers, UniProt database, etc., the Project HOPE server foresees the structural effects of nsSNPs [65].

### Comparative modeling and evaluation of mutated 3D structures

The three-dimensional (3D) model of the mutant proteins was obtained through comparative modeling in Modeller 10.2 [https://salilab.org/modeller/] standalone software. The AlphaFold structure of wild-type protein was used as a template for generating altered protein structure. A comprehensive optimization protocol was followed to ensure high accuracy. The optimization schedule was modified to give less weight to soft-sphere restraints, with the scaling factor set to 0.7. For the optimization configuration, the Variable Target Function Method (VTFM) was set to a thorough schedule with a maximum of 300 iterations, while Molecular Dynamics (MD) with Simulated Annealing (SA) was configured for thoroughness. Additionally, the entire optimization process was repeated twice, with the objective function limit set to 1×10^6^. The energy of the model was minimized according to the default system that constructs a scoring function from the available data and then minimizes it. All mutant structures were generated using the same default seed value (-8321) to ensure consistency in the structural generation process [66]. After completion of the 3D model generation, PyMOL 2.5 [https://pymol.org/2/] software was utilized to analyze each mutant structure’s root mean square deviation (RMSD) value. By superimposing native and mutant structures, this tool forecasts the RMSD value, which aids in identifying the closest related structural analog. Then, the structure validation of the 3D model of each mutant protein was analyzed through the SAVES [https://saves.mbi.ucla.edu/] server.

### Analysis of secondary structure, domains, and PTM sites

To analyze the secondary structure, all ten variant sequences, along with the native sequence, were evaluated using PDBsum [https://www.ebi.ac.uk/thornton-srv/databases/pdbsum/]. Mutation 3D [http://mutation3d.org/] was utilized to assess the arrangements of SNPs on protein models or structures and to look for the functional domain information of the SNP positions [67]. Through the complete-linkage clustering procedure, this tool also identifies clusters of amino acid substitutions in protein structure, which indicates the positions that have the most impact on the structure of a protein. Lastly, MusiteDeep [https://www.musite.net/] was employed to predict the putative PTM sites in RTEL1 protein. Utilizing a deep learning-based algorithm and depending on the confidence threshold, with a default cut-off of 0.5, MusiteDeep predicts and identifies the desired PTM sites in the sequence [68].

### Prediction of evolutionary conservation and surface accessibility

The evolutionary conserved amino acid position in RTEL1 protein was interpreted using ConSurf [https://consurf.tau.ac.il/consurf_index.php] web server [69]. In this server, the evolutionary profile is computed by searching for homologous sequences and multiple sequence alignment (MSA), then generating a phylogenetic tree using a neighbor-joining algorithm. Moreover, through the Bayesian method [70], this tool enumerates a site-specific conservation score from 1 to 9, with 9 representing a highly conserved position [71]. NetSurfP-2.0 [http://www.cbs.dtu.dk/services/NetSurfP/] is a sequence-based web server that employs convolutional and long short-term memory neural network architecture to predict structural features such as surface accessibility, structural disorder, and secondary structure for each amino acid position [72]. To assess the surface accessibility of each amino acid residue of the RTEL1 protein, the protein sequence was run within the default parameter in the NetSurfP-2.0 server. A phylogenetic tree of the ten closest matches to the human RTEL1 protein, determined by BLASTp search, was constructed in MEGA11 software using the maximum likelihood technique and a bootstrap parameter of 1000 [73]. This enables us to elucidate the evolutionary relationship of the RTEL1 protein. The tree was then visualized using the Iroki web server [https://www.iroki.net/] [74].

### Cancer susceptibility prediction

The oncogenic susceptibility of the selected nsSNPs was evaluated through CScape [http://cscape.biocompute.org.uk/] and CanSAR.ai [https://cansar.ai/]. Following a statistical approach, CScape can predict the likelihood of a mutation to be cancer-causing with a 91% balanced accuracy in coding regions of the genome [75]. The server takes the mutations list using the format chromosome, position, reference base, and mutant base and returns the result as p-values (probability scores) between [0, 1], with values above 0.5 projected to be harmful and values below 0.5 predicted to be neutral or benign. P-values close to the extremes (0 or 1) are the highest-confidence predictions that yield the highest accuracy. Next, CanSAR.ai was used to find the association of specific SNPs with different cancer types previously identified in different studies. This tool is an integrative translational research knowledgebase for cancer with the integration of multidisciplinary data [76].

### Interatomic interaction prediction

The interatomic interaction was predicted by implementing several programs of PyMOL 2.5 software [https://pymol.org/2/], which helps to visualize the change in atomic interaction in amino acid residues due to any single mutation. The polar contacts of selected residue with other atoms were searched for, and the distance between the atoms was measured.

### Molecular docking analysis

Using the HDOCK [http://hdock.phys.hust.edu.cn/] web server, molecular docking with telomeric DNA corresponding to PDB ID 1W0U [77] was performed on the selected most harmful mutant structures and the native structure. HDOCK server predicts the binding complexes between protein and nucleic acid by following the hybrid docking approach [78, 79]. For the input molecule in the server, protein structure (wild type and mutant) and DNA structure were provided as receptor molecule and ligand molecule, respectively.

The literature shows that the HHD2 (Harmonin Homology Domain 2) domain of RTEL1 interacted directly with DNA [80]. Therefore, to specify the binding site, the positions of the HHD2 domain (A1059, V1060, S1061, A1062, Y1063, L1064, A1065, D1066, A1067, R1068, R1069, G1075, S1077, Q1078, L1079, L1080, A1081, A1082, T1084, K1087, D1090, and D1134) mentioned in the literature were used here as a receptor binding site residues and the TTAGGG motif and its complementary sequence positions were selected from both strands (chain C and chain D) of DNA for ligand binding site residue. From the provided HDOCK result, docked models were chosen based on the following criteria: smaller docking score, confidence score ≥0.5, and smaller RMSD value and subjected to DNAproDB [https://dnaprodb.usc.edu/] to visualize the interaction patterns that each complex formed [81, 82].

### 5’ and 3’ UTR non-coding SNPs assessment

To evaluate the functional effects of the filtered-out non-coding SNPs from the Ensemble database, RegulomeDB [https://regulomedb.org/regulome-search] was used. With the combinatorial uses of numerous high-throughput experimental datasets, this server detects non-coding SNPs with possible regulatory roles [83]. Lastly, in order to determine whether any of the non-coding SNPs were found in the seed regions and target sites of microRNAs (miRNA), the PolymiRTS [https://compbio.uthsc.edu/miRSNP/] database was searched [84].

**Fig 1:**
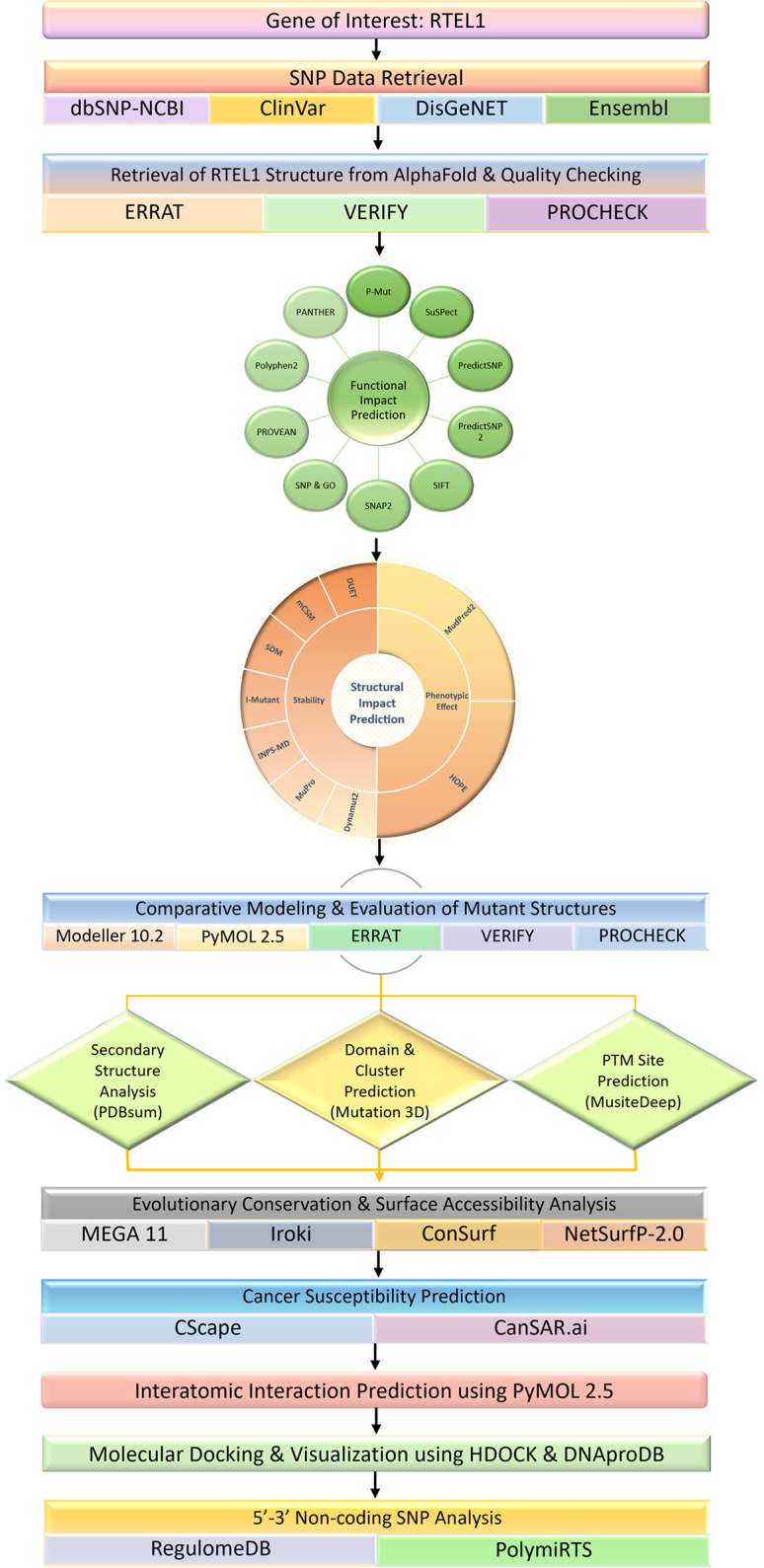
Schematic illustration of the workflow of the study along with the tools and software used during the investigation process.

A schematic representation of the workflow of this study is provided in Fig 1.

## Results

### SNP annotation

The Single Nucleotide Polymorphism data about the human RTEL1 gene was retrieved from the NCBI dbSNP database. Among the 20734 SNPs from the search result, 25 are inframe deletions, 17554 are in the intronic region, 1392 are missense (non-synonymous), 2522 are non-coding variants, and 781 are synonymous. For this study, only the nsSNPs or missense SNPs (a total of 1392) were filtered out from the dbSNP database. After removing redundancy, 347 SNPs and 23 SNPs were filtered out from ClinVar and DisGeNET databases, respectively, but all were found to be annotated in the NCBI dbSNP database. Therefore, in total, 1392 nsSNPs, which occurred in 1383 unique positions, were considered for subsequent analysis. After being collected from the Ensemble database, the non-coding SNPs located at the 5’-3’ UTR region were filtered out based on a global minor allelic frequency (MAF) value between 0.01 and 0.5.

### Assessment of RTEL1 protein structure

**Fig 2:**
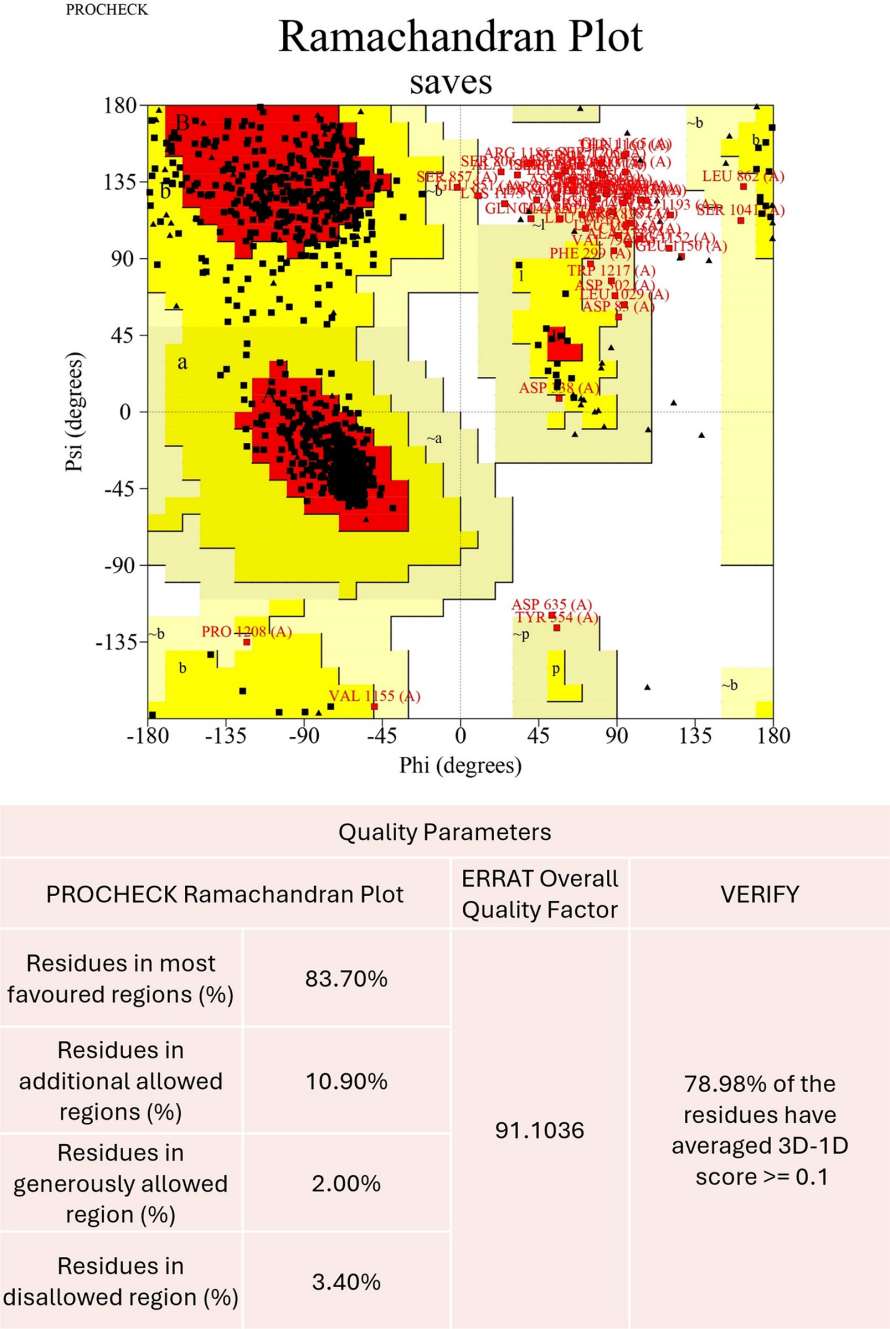
Ramachandran plot of the AlphaFold structure of RTEL1 protein from PROCHECK and quality parameters derived from SAVES server.

The tertiary structure of the protein determines its properties and capacity for interacting with ligands. As no full-length crystal structure was found in the protein data bank for human RTEL1 protein, the AlphaFold structure of RTEL1 protein was taken from UniProt. The structure was validated using the SAVES server, where ERRAT provided 91.1036 for the overall quality factor and Verify-3D revealed that 52.83% of the residues have an average 3D-1D score of 0.2. The Ramachandran plot, available in PROCHECK, was utilized to evaluate further the quality of the 3D protein structure (Fig 2). The plot from the AlphaFold model revealed that 93.7% of the residues are in the favoured region, 10.9% are in the additional allowed region, 2.0% are in the generously allowed region, and 3.4% are in the disallowed region. The general conclusions drawn from the results mentioned above pointed to the good quality of our protein structure, which allowed it to be used in subsequent investigations.

### Determination of functional consequences of RTEL1 nsSNPs

The functional impact of nsSNPs on RTEL1 has been assessed using ten tools. SIFT predicted 441 as damaging, of which 88 had a low confidence score. Therefore, 353 remained the most functionally detrimental after eliminating the redundancies. Out of the submitted 1392 nsSNPs, the PROVEAN server identified 489 as potentially harmful. PolyPhen-2 and Panther anticipated 386 and 579 as probably damaging ones, respectively. Moreover, SuSPect provides a list of scores ranging from 0–100 for each variant that is likely to be disease-causing, and the recommended cutoff is 50 for the most deleterious ones. Therefore, 72 disease-causing variants with a score of ≥50 were chosen from the SuSPect output. PredictSNP integrates the results of six (MAPP, PhD-SNP, Polyphen1, Polyphen2, SIFT, SNAP) best-performing tools, while PredictSNP2 combines the results of five top tools (CADD, DANN, FATHMM, FunSeq2, GWAVA) and gives a consensus score. Only the consensus score from both tools was considered, where PredictSNP and PredictSNP2 identified 309 and 364 as deleterious, respectively. In addition, 280 nsSNPs were found to be pathological in P-Mut, 505 nsSNPs were predicted to be impactful in SNAP2, and 166 nsSNPs were disease-associated in SNP and GO.

Among 1392 nsSNPs, 43 were deemed functionally harmful by all 10 different tools, and the remaining SNPs were assumed to be neutral in at least one of these tools. So, considering only the common variants predicted by all ten tools, 43 nsSNPs (S1 Table) were selected for further analysis.

### Determination of structural impact of RTEL1 nsSNPs

To determine the structural impact of nsSNPs on RTEL1 protein, the filtered nsSNPs from the upstream analysis were subjected to nine different tools. Among these nine tools, seven were utilized for predicting stability changes, and two were used for phenotypic effect prediction.

The change in the structural stability of RTEL1 protein due to the introduction of point mutations was predicted through seven bioinformatics-based web tools. The 43 deleterious nsSNPs were run to check the structural stability of proteins in the DUET server, including the mCSM and SDM results. mCSM, SDM, and DUET predicted 36, 30, and 33 nsSNPs as destabilizing for RTEL1 protein, respectively. To increase the accuracy of our predictions of changes in protein stability caused by single AA mutations, all 43 variants were analyzed through I-Mutant, INPS-MD, Mupro, and Dynamut2. I-Mutant and MuPro predicted 34 and 41 nsSNPs as stability-decreasing. Moreover, 40 nsSNPs with a negative ΔΔG score were considered destabilizing in the INPS-MD result. Lastly, by combining the structure or NMA-based prediction (ΔΔG ENCoM) and vibrational entropy change (ΔΔS ENCoM) between mutant and wild-type structures, Dynamut2 provides the ΔΔG prediction score for each amino acid substitution. Here, 36 nsSNPs were predicted to be destabilizing by Dynamut2.

Combining the findings from seven tools, 13 nsSNPs were identified unanimously by all of these tools as extremely detrimental based on their effects on the structural stability of proteins (Table 1).

**Table 1:** Predication of the destabilizing effect of nsSNPs determined by 7 different web tools.

Serial No.	nsSNPs	mCSM		SDM		DUET		I-Mutant2		INPS-MD		MuPro		Dynamut2	
		Stability Change	ΔΔG in kcal/mol	Stability Change	ΔΔG in kcal/mol	Stability Change	ΔΔG in kcal/mol	Stability	RI	Stability Change	ΔΔG in kcal/mol	Stability Change	ΔΔG in kcal/mol	Stability Change	ΔΔG in kcal/mol
1	F15L	Decrease	-1.3	Decrease	-0.05	Decrease	-1.2	Decrease	9	Decrease	-1.99	Decrease	-0.31	Decrease	-1.49
2	M25V	Decrease	-1.47	Decrease	-0.88	Decrease	-1.23	Decrease	5	Decrease	-1.89	Decrease	-0.88	Decrease	-0.81
3	R141Q	Decrease	-0.9	Decrease	-0.88	Decrease	-0.84	Decrease	9	Decrease	-1.32	Decrease	-0.47	Decrease	-0.67
4	A252V	Decrease	-0.34	Decrease	-0.64	Decrease	-0.29	Decrease	1	Decrease	-1.06	Decrease	-0.47	Decrease	-1.23
5	G480R	Decrease	-0.89	Decrease	-1.95	Decrease	-0.88	Decrease	8	Decrease	-0.6	Decrease	-0.85	Decrease	-0.86
6	F559L	Decrease	-1.82	Decrease	-2.25	Decrease	-2.16	Decrease	9	Decrease	-1.52	Decrease	-1.55	Decrease	-1.6
7	R639H	Decrease	-2.46	Decrease	-0.42	Decrease	-2.51	Decrease	7	Decrease	-1.1	Decrease	-1.29	Decrease	-0.88
8	G645D	Decrease	-2.13	Decrease	-1.87	Decrease	-2.25	Decrease	6	Decrease	-0.91	Decrease	-0.87	Decrease	-1.78
9	R697Q	Decrease	-1.68	Decrease	-1.88	Decrease	-2.06	Decrease	8	Decrease	-0.85	Decrease	-0.71	Decrease	-2.42
10	R700Q	Decrease	-1.02	Decrease	-2.27	Decrease	-1.42	Decrease	9	Decrease	-1.06	Decrease	-1.01	Decrease	-0.74
11	G706R	Decrease	-0.85	Decrease	-2.32	Decrease	-0.79	Decrease	7	Decrease	-0.58	Decrease	-1.09	Decrease	-0.96
12	R729C	Decrease	-1.06	Decrease	-0.29	Decrease	-0.94	Decrease	7	Decrease	-0.16	Decrease	-0.73	Decrease	-0.88
13	H960R	Decrease	-1.52	Decrease	-1.55	Decrease	-1.6	Decrease	3	Decrease	-0.1	Decrease	-0.69	Decrease	-1.47

The phenotypic effects of 13 functionally damaging SNPs were computed using MutPred2 and Project HOPE. Together with the P-value and probability score, some predictions made using MudPred2 were loss or gain of allosteric site, catalytic site, helix, relative solvent accessibility, increase in various types of modification such as transmembrane protein, DNA, ligand, metal binding, or ordered interface, etc. Besides that, a MutPred2 score was given, with a cutoff of 0.50, determining the overall probability of pathogenicity. The score goes from 0 to 1, and as the score rises, it becomes more likely that the SNP-induced alterations can influence the molecular mechanism of disease. Except for F559L, all the other nSNPs were identified as having higher pathogenic potential (Table 2).

**Table 2:** Analysis of the phenotypically damaging nsSNPs predicted by MutPred 2 and Project HOPE.

Serial No.	nsSNPs	HOPE		MutPred2	
		Pathogenicity based on Conservancy	Phenotypic Effects	Pathogenicity Score	Predicted Molecular Mechanism
1	F15L	probably damaging	A smaller mutant amino acid might lead to loss of external protein interactions.	0.773	Altered Ordered interface; Loss of Relative solvent accessibility; Loss of Allosteric site at F13; Altered Metal binding
2	M25V	probably damaging	An empty space in the protein’s core could result from a smaller mutant amino acid.	0.841	Altered Ordered interface
3	R141Q	probably damaging	Loss of charge in a mutant amino acid may disrupt interactions with other molecules.	0.774	Loss of Allosteric site at R141; Loss of Catalytic site at R141
4	A252V	probably damaging	A larger mutant amino acid on the protein surface might disturb molecular interactions.	0.78	Loss of Catalytic site at E251; Gain of Allosteric site at A252; Altered Metal binding
5	G480R	probably damaging	A bigger, charged mutant amino acid could repel neighboring residues and disturb protein structure.	0.925	Loss of Catalytic site at T481; Altered Ordered interface; Gain of Helix; Gain of Allosteric site at G480; Altered Metal binding; Altered Transmembrane protein
6	R639H	probably damaging	Loss of charge and a smaller mutant amino acid might lead to fewer external interactions.	0.839	Altered Metal binding; Loss of Allosteric site at R639; Loss of Relative solvent accessibility; Gain of Loop; Gain of Catalytic site at D635
7	G645D	probably damaging	Introduction of a charge by a larger mutant amino acid could cause protein folding problems.	0.956	Altered Metal binding; Gain of Catalytic site at G645; Gain of Allosteric site at G645; Gain of Relative solvent accessibility
8	R697Q	probably damaging	A smaller mutant amino acid could lead to loss of interactions due to charge and size differences.	0.913	Loss of Allosteric site at R697; Gain of Catalytic site at R700; Altered Disordered interface; Altered Metal binding; Altered DNA binding
9	R700Q	probably damaging	The mutation causing loss of charge and a smaller amino acid might reduce external interactions.	0.897	Altered Metal binding; Loss of Allosteric site at H701; Altered Ordered interface; Altered Disordered interface; Gain of Catalytic site at R700; Altered DNA binding
10	G706R	probably damaging	A larger mutant amino acid introducing a charge could lead to protein folding issues and structural disruption.	0.917	Altered Metal binding; Loss of Strand; Gain of Allosteric site at H701; Altered Ordered interface; Altered Disordered interface; Loss of Catalytic site at D704
11	H960R	probably damaging	A larger mutant amino acid introducing a charge might cause repulsion and structural issues.	0.721	Altered Metal binding

Additionally, the mutations were submitted to HOPE for analysis. According to HOPE results, 9 of the 13 mutant amino acids differed in charge, one differed in the level of hydrophobicity, and all 13 mutant residues were predicted to differ in size from the wild-type residue. These differences in size, charge, and hydrophobicity can interfere with the nearby amino acid residues’ interactions and protein folding. Aside from these, amino acid substitution also impacts numerous other attributes. For example, substitutions involving glycine may disrupt protein conformation by interfering with the flexibility that glycine imparts due to its greater conformational freedom. HOPE also provides a result of pathogenicity based on conservancy where R729C predicted as less damaging.

Finally, 11 nsSNPs were repeatedly recognized by MutPred2 and Project HOPE web server as being particularly harmful based on their effects on protein phenotype (Table 2). These SNPs were found to induce a decrease in protein stability and negatively impact other properties.

### Three-dimensional structure prediction for mutant proteins

To investigate whether the selected nsSNPs cause any alteration in the resultant protein, comparative 3D modelling and structural comparison between native and mutant structures were carried out through Modeller 10.2, followed by PyMOL 2.5 software. The wild-type amino acid residues in the selected deleterious SNP positions in the RTEL1 protein sequence were replaced with the mutant amino acid to generate the sequence for each variant. The mutated protein sequence was then utilized in Modeller 10.2 to develop the 3D structure for each variant using the AlphaFold structure as a template.

Next, the RMSD values of the mutant models were examined in PyMOL 2.5 to investigate structural similarity between the native and mutant protein structures. All the mutant models were observed to have a high RMSD value (Table 3) when superimposed over the native structure (S1 Fig). Also, the results of ERRAT, VERIFY, and PROCHECK Ramachandran Plot from the SAVES server validated the quality of the mutant models. As the larger RMSD value demonstrates greater deviation between wild-type and mutant structures, all 11 nsSNPs were considered for the following investigation.

**Table 3:** RMSD values of 11 mutated RTEL1 protein models and the quality parameters of each structure generated through PyMOL and SAVES server, respectively.

Serial No.	Mutation	RMSD^a^ Values	Quality Parameters					
			PROCHECK Ramachandran Plot				ERRAT Quality Factor	VERIFY (percentage of the residues have averaged 3D-1D score > = 0.1)
			Residues in most favoured regions (%)	Residues in additional allowed regions (%)	Residues in generously allowed region (%)	Residues in disallowed region (%)		
1	F15L	0.471	94.00%	5.10%	0.60%	0.40%	82.613	75.48%
2	M25V	0.393	94.20%	4.70%	0.80%	0.40%	86.0119	78.76%
3	R141Q	0.455	94.20%	5.10%	0.50%	0.30%	84.6693	76.84%
4	A252V	0.685	94.50%	4.70%	0.70%	0.20%	84.7913	78.08%
5	G480R	0.437	94.00%	5.40%	0.20%	0.40%	80.0399	76.61%
6	R639H	0.396	94.40%	4.90%	0.40%	0.40%	83.9161	73.90%
7	G645D	0.526	93.70%	5.40%	0.50%	0.40%	83.4325	75.71%
8	R697Q	0.511	94.50%	4.70%	0.50%	0.40%	84.1584	78.53%
9	R700Q	0.58	94.80%	4.50%	0.50%	0.30%	82.9681	80.11%
10	G706R	0.418	94.20%	4.90%	0.80%	0.20%	84.4554	76.16%
11	H960R	0.574	93.50%	5.80%	0.40%	0.30%	80.8448	79.10%

^a^RMSD: Root Mean Square Deviation

### Investigation of the impact of nsSNPs on secondary structure, domains & clusters, and PTM sites

**Fig 3:**
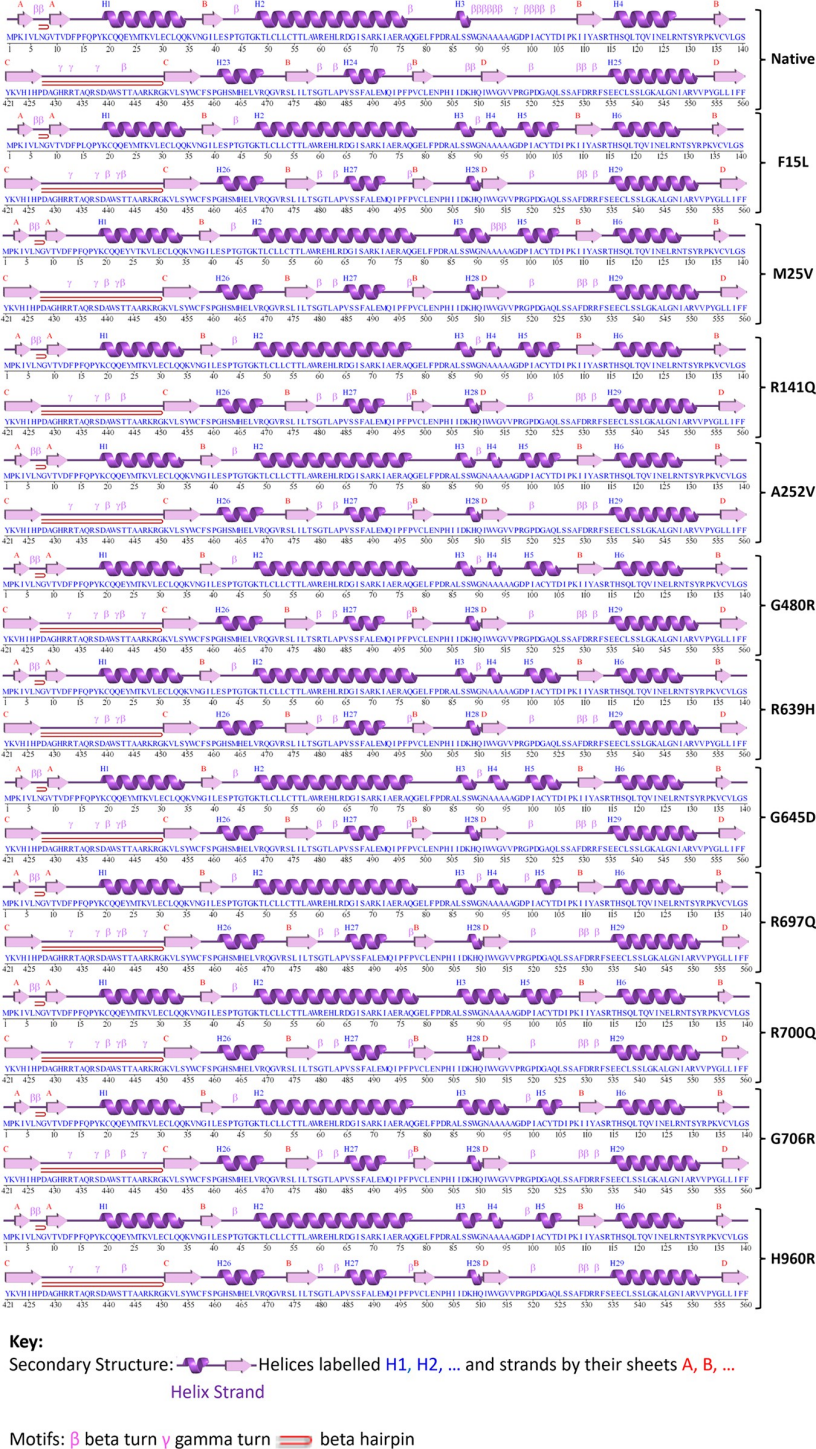
Analysis of wild-type and mutated RTEL1 protein secondary structures using PDBsum. It displays the changes brought on by nsSNPs in terms of alpha helices, beta strands, and other patterns.

The prediction of secondary structure conformation of RTEL1 and 11 mutants was performed in the PDBsum web tool. The tool’s output found that both the wild-type and mutant structures have the same number of strands, sheets, beta hairpins, and beta alpha beta units. Apart from the number of helix-helix interactions, which remained the same in the native and M25V mutant structure, the number of helices and helix-helix interactions were increased in mutant structures compared to the native structure. Additionally, in all mutant structures, the amount of beta and gamma turns was reduced, as shown in Table 4. Furthermore, in the native structure, positions W89 to D105 had many closely packed beta turns, whereas, in the mutant structures, this varied widely (either absent or 2/3 beta turns were present). Besides, F15L, M25V, A252V, G480R, R639H, G645D, R697Q, and R700Q mutants showed more tightly packed beta and gamma turns after position A429 than R141Q, G706R, and H960R mutants (Fig 3).

**Table 4:** ProMotif information of the secondary structure of native and mutant protein.

Protein	Sheets	beta alpha beta units	beta hairpins	strands	helices	helix-helix interacts	beta turns	gamma turns
Wild-type	4	3	3	20	53	74	67	14
F15L	4	3	3	20	58	80	41	7
M25V	4	3	3	20	60	74	43	10
R141Q	4	3	3	20	58	76	40	9
A252V	4	3	3	20	58	75	40	9
G480R	4	3	3	20	57	75	43	9
R639H	4	3	3	20	58	75	41	7
G645D	4	3	3	20	58	79	40	8
R697Q	4	3	3	20	58	77	41	10
R700Q	4	3	3	20	57	76	41	10
G706R	4	3	3	20	58	81	40	8
H960R	4	3	3	20	58	77	40	8

**Fig 4:**
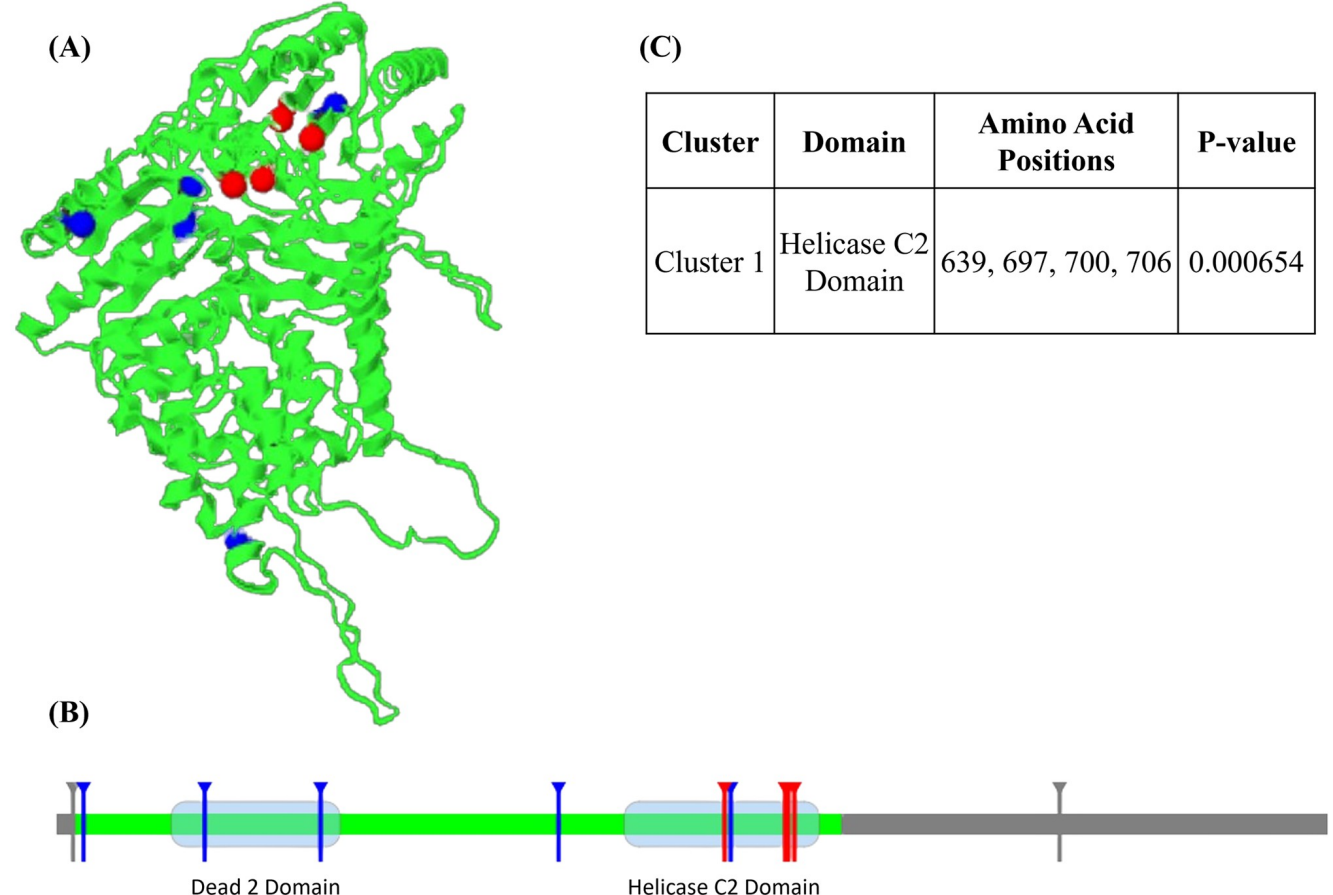
Domain & cluster information of nsSNPs represented in linear and 3D protein model. (A) 3D protein model represents atomic coordinates based on the corresponding ModBase structure where substitutions in the cluster are shown in red spheres. (B) Helicase C2 (right) and Dead2 (left) domains are indicated as a light blue transparent box in the highlighted green region of the linear model, and the position of amino acid substitutions is portrayed in vertical lines. (C) Mutation cluster prediction from Mutation 3D (upper right side).

Mutation 3D was used to predict mutant positions in domains and clusters, and the tool predicted two domains based on the submitted data. Dead 2 domain (111–272) contains R141Q, and A252V mutants, and Helicase C2 domain (545–731) contains R639H, G645D, R697Q, R700Q, and G706R, mutants. Moreover, the tool projected ModBase model, featuring one cluster, which housed R639H, R697Q, R700Q, and G706R (Fig 4). According to the findings of Mutation3D, four mutants were found to be part of a cluster, indicating that these mutations may have the greatest impact on the protein structure. Even though the rest of the mutants were not predicted to form clusters, we kept all of them for further analysis as those were predicted to be deleterious in former investigations.

To predict the potential PTM sites in RTEL1 and the effects of SNPs on PTM sites, MusiteDeep was used. A total of 8 types of 74 PTM sites were predicted for the protein sequence. Among all the selected deleterious SNPs, only the R639 position was predicted to be in a methylation site. Studies have linked methylation to fine-tuning various biological processes, resulting in the formation of numerous diseases [85]. Thus, amino acid alteration in position 639 can be anticipated to result in PTM impairment.

### Analysis of evolutionary relationship of RTEL1 protein and conservation profile & surface accessibility of nsSNPs

Despite the evolutionary change, amino acid residues essential for various biological functions, including genome integrity, typically persist. Because of this, it is frequently believed that the degree of residue conservation indicates how crucial a location is to preserve the stability and functionality of a protein. In this regard, the conservation profile and surface accessibility of the 11 nsSNPs were analyzed through the ConSurf and NetSurfP web tools, along with inspecting the evolutionary relationship of RTEL1 protein using MEGA 11 software.

**Fig 5:**
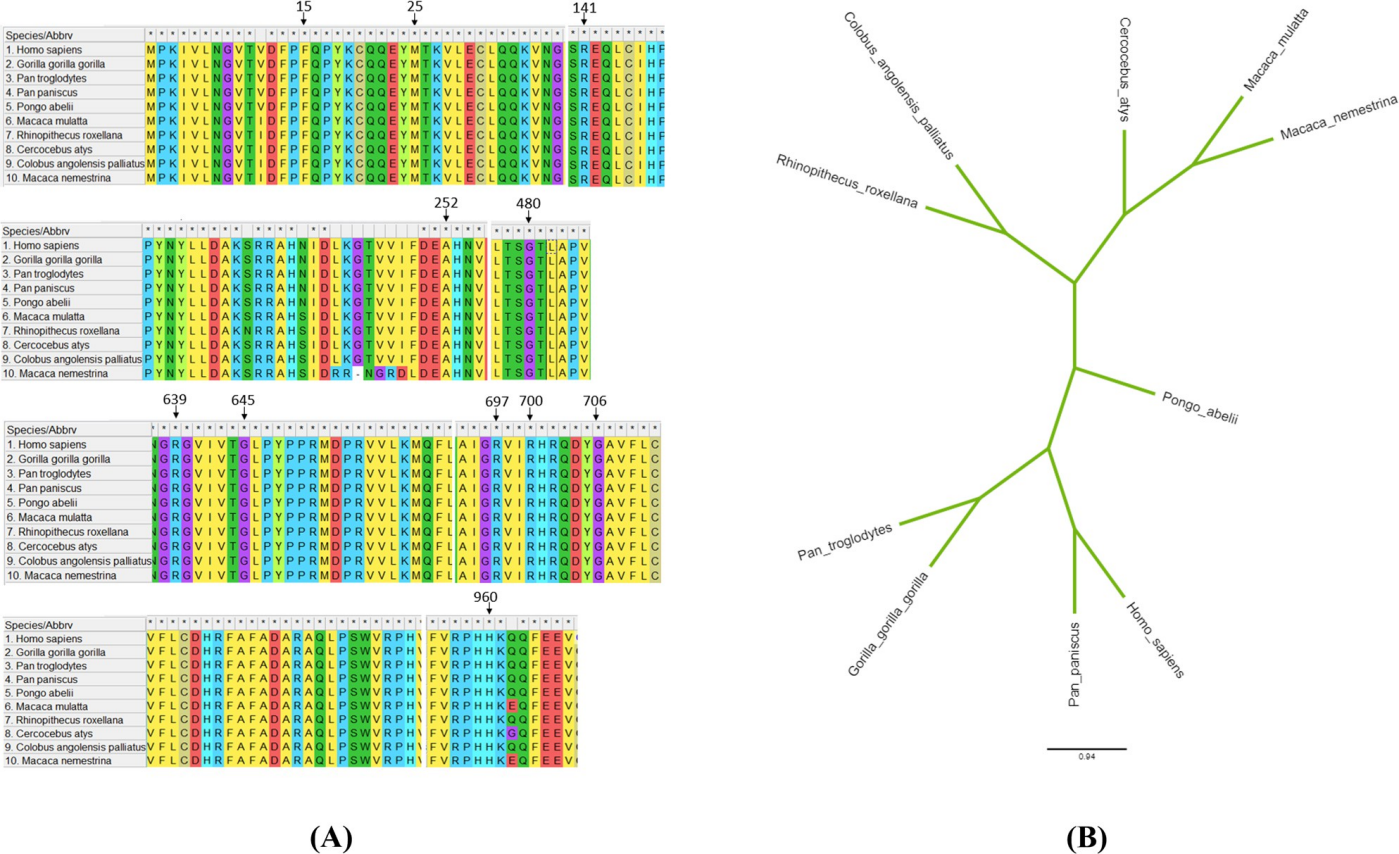
Assessment of the evolutionary relationship of RTEL1. (A) Evolutionary conservancy of 11 nsSNPs analyzed through multiple sequence alignment. (B) Graphical depiction of the evolutionary relationship of human RTEL1 with its closest relatives.

The MEGA 11 program was used to analyze the conservation of the selected 11 SNP positions in 10 different species, along with phylogenetic analysis to determine the evolutionary relationships between these species. Then, the tree was displayed by Iroki to examine evolutionary conservation. According to the findings, all amino acid positions are conserved among these ten species. Moreover, *Pan paniscus*, *Pan troglodytes*, and *Gorilla gorilla* are the three species that have been found to share the largest genetic similarity with the human RTEL1 protein (Fig 5). So, according to the phylogenetic tree, it can be said that the RTEL1 protein is conserved in primates.

**Fig 6:**
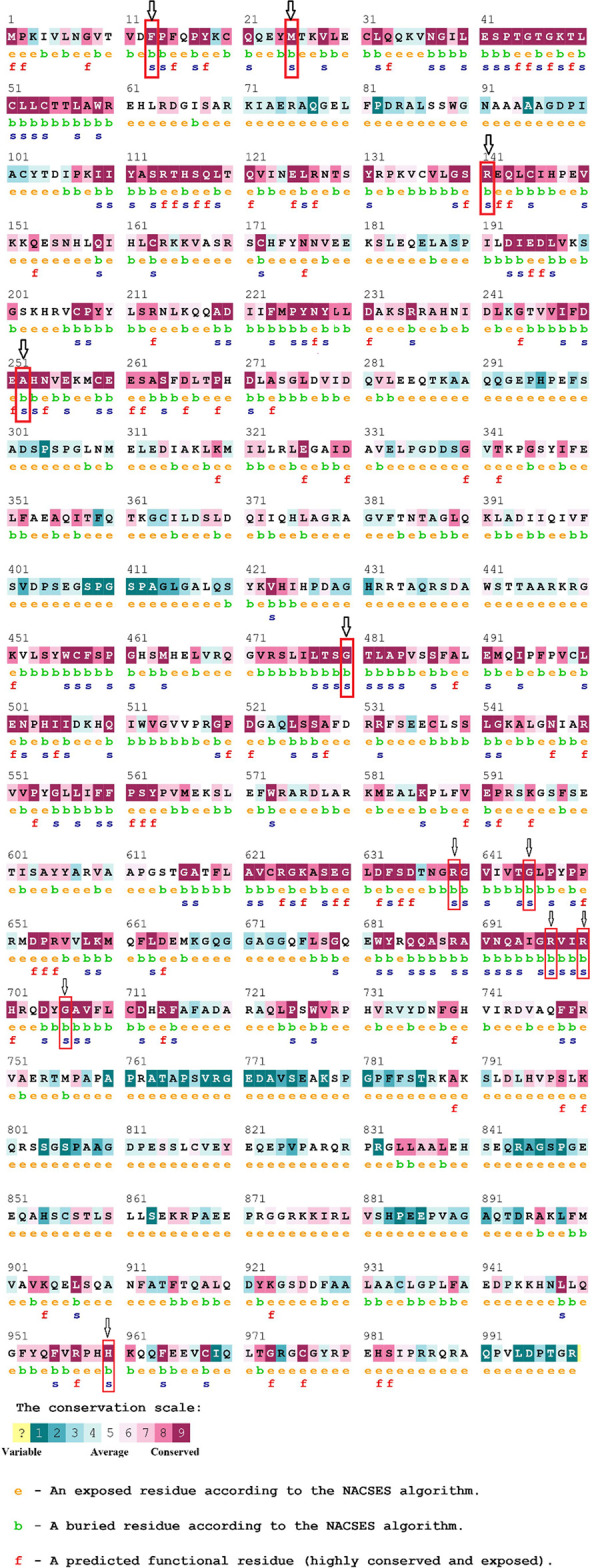
Evolutionary conservation profile of RTEL1 protein from ConSurf web server. All of the nsSNPs identified as harmful belonged to highly conserved regions in the RTEL1 protein.

To determine the conserved positions in the amino acid sequence of RTEL1 protein, the ConSurf server was used. Using the Bayesian approach, the ConSurf online browser assessed the degree of conservation of each protein residue along with identified potential structural and functional residues. The result showed all eleven residues filtered out from the upstream study are structural (buried) residues, with a highly conserved profile. Moreover, on the conservation scale of 1–9, ten positions exhibit the highest conservation profile with a conservation score of 9, and one position (F15) has a high level of conservation with a conservation score of 8 (Fig 6).

With the percentage scores, NetSurfP-2.0 estimated the surface accessibility of each amino acid site of the RTEL1 protein. The relative surface accessibility of each position in the amino acid sequence was predicted at a threshold of 25%, which meant that amino acid residues with scores of more than 25% were expected to be exposed, whilst residues with scores of less than 25% were assumed to be buried. Among eleven selected positions, R141, and R639 each received a score of more than 25%. Therefore, these amino acid residues were anticipated to be exposed, while the remaining 9 locations were expected to be in the buried zone, scoring less than 25% (Table 5).

**Table 5:** Surface accessibility result of 11 nsSNPs generated by NetSurf2.0.

Class assignment	Amino acid	Amino acid number	Relative Surface Accessibility
Buried	F	15	0.116
Buried	M	25	0.012
Exposed	R	141	0.315
Buried	A	252	0.034
Buried	G	480	0.15
Exposed	R	639	0.287
Buried	G	645	0.061
Buried	R	697	0.145
Buried	R	700	0.17
Buried	G	706	0.005
Buried	H	960	0.097

While modification of amino acids in a highly conserved position can possibly be more harmful than in any non-conserved position, it is also possible for functional variants to exist without causing harm. Additionally, the residues in the buried or exposed zone can also potentially hamper the structure of the proteins and their interaction. Therefore, based on the outcomes of the tools, it can be said that the 11 selected nsSNPs may significantly impact the RTEL1 protein.

### Prediction of high-risk nsSNPs with cancer susceptibility

The initial evaluation of the oncogenic potential of 11 nsSNPs was performed in CScape. All of the mutations were predicted to be deleterious; among them, five (R639H, G645D, R697Q, R700Q, G706R) demonstrated the highest degree of confidence of being oncogenic (Table 6). Next, these mutations were searched in canSAR.ai, and from the search result, the association of G480R, and G706R mutations was found with liver, and endometrial cancer, respectively [86].

**Table 6:** The cancer susceptibility predictions, scores, and association with different types of cancer of the selected nsSNPs determined by CScape and canSAR.ai.

CScape						canSAR.ai association
Chromosome	Position	Ref. Base	Mutant Base	Coding Score	Prediction	
20	62290800	C	A	0.580519	oncogenic	
20	62290828	A	G	0.701031	oncogenic	
20	62293925	G	A	0.876857	oncogenic	
20	62303964	C	T	0.84255	oncogenic	
20	62319080	G	C	0.856576	oncogenic	Liver Cancer
20	62320892	G	A	0.910291	oncogenic	
20	62320910	G	A	0.890656	oncogenic	
20	62321167	G	A	0.964741	oncogenic	
20	62321176	G	A	0.913183	oncogenic	
20	62321193	G	A	0.920325	oncogenic	Endometrial Cancer
20	62324523	A	G	0.684018	oncogenic	

### Prediction of interatomic interaction

**Fig 7:**
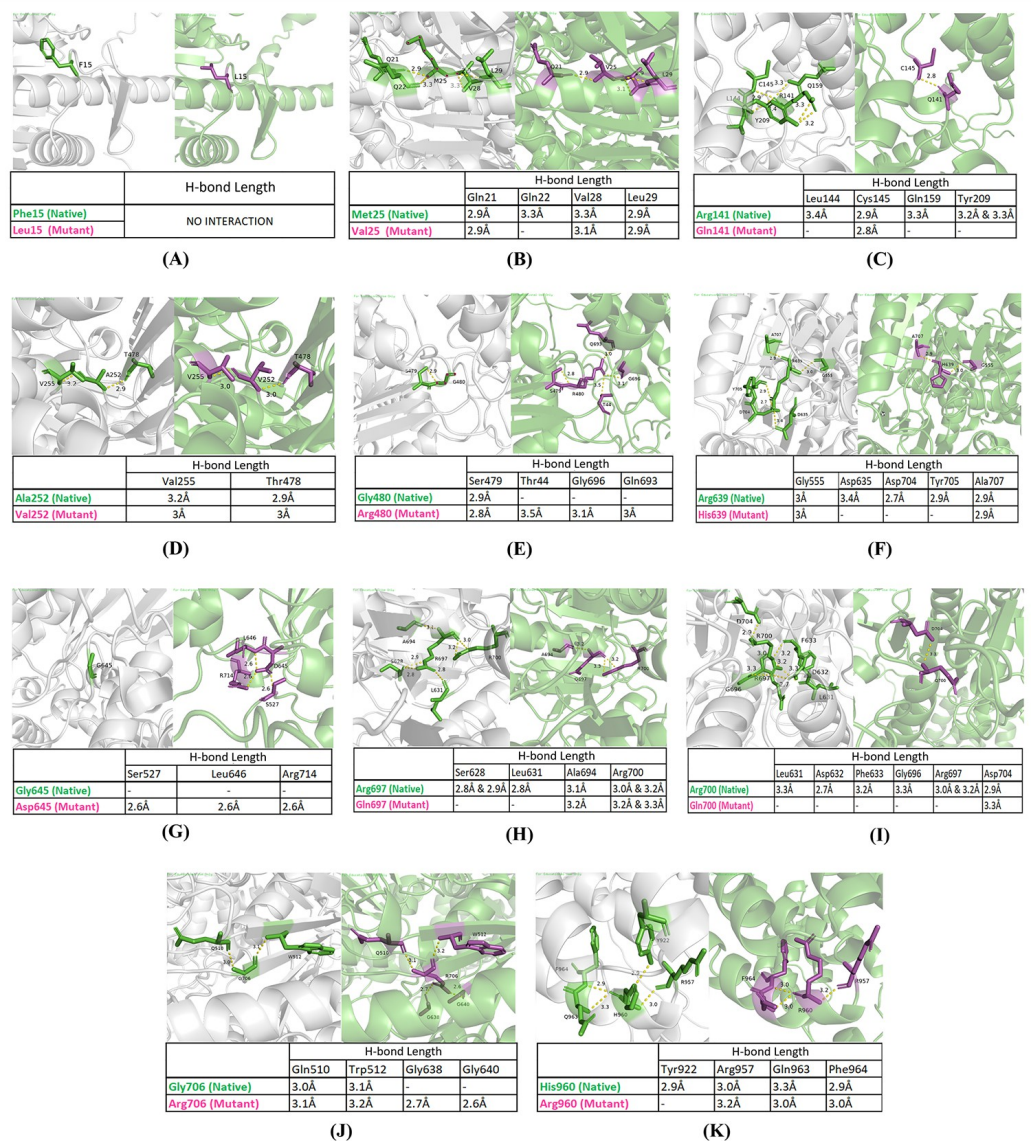
Analyses of the effect of nsSNPs on the interaction pattern with the neighboring residues. The distance from nearby amino acid atoms in (A) F15L, (B) M25V, (C) R141Q, (D) A252V, (E) G480R, (F) R639H, (G) G645D, (H) R697Q, (I) R700Q, (J) G706R, and (K) H960R mutant structure are visualized using PyMOL2.5.

In the case of the substitution of phenylalanine with leucine at position 15, no alteration in interatomic interaction was observed. In the native structure, methionine at position 25 forms H -bonds with four nearby residues Gln21, Gln22, Val28, and Leu29, whereas due to the substitution of methionine with valine, the number of interacting residues decreased to three, and the distance remained quite similar to that of the wild-type residue. For the substitution of arginine with glutamine at position 141, only one H-bond with Cys145 remained intact in the mutant, with the distance being decreased to 2.8 Å, and the rest of the interactions were eliminated. When comparing wild-type and mutant amino acids, it was found that the A252V mutation did not significantly alter the H-bond pattern and that the distance between the neighboring residues (Val255 and Thr478) remained nearly unchanged. The H-bond distance between Gly480 and the nearby Ser479 residue was 2.9 Å, as shown in Fig 7E. Due to glycine being replaced with arginine, the bond distance was reduced to 2.8 Å, and three additional H-bonds with neighboring Thr44, Gly696, and Gln693 were introduced in the mutant structure. The mutation G645D formed new H-bonds with Ser527, Leu646, and Arg714 each having a length of 2.6 Å.

Moreover, the H-bond distance between Arg639 (wild type) and nearby Gly555, Asp635, Asp704, Tyr705, and Ala707 residues was 3 Å, 3.4 Å, 2.7 Å, 2.9 Å, and 2.9 Å, whereas for His639 (mutant), the values were 3 Å and 2.9 Å for Gly555 and Ala707, respectively, and the rest of the H-bonds were not observed to persist in the mutated protein structure. Furthermore, when arginine was replaced with glutamine, the structure relaxed because five of the six H-bonds observed in the wild-type amino acid with Leu631, Asp632, Phe633, Gly696, and Arg697 were eliminated in the mutant amino acid, while the remaining H-bond (Asp704) showed the slightest increase (2.9 Å to 3.3 Å) in distance. On the other hand, the mutant at position 960 had little effect on interaction, where the H-bond with Tyr922 was canceled out, along with minimal fluctuation in other H-bonds with neighboring atoms (Fig 7K). Among the other two mutations, one showed complete elimination of two H-bonds while the other showed introduction of two new H-bonds. In the case of R697Q, H-bond with Ser628 and Leu631 was eliminated, and the interacting distance with both Ala694 and Arg700 increased by at least 1 Å. Finally, when glycine was switched out for arginine at position 706, two new H-bonds with Gly638 and Gly640, at distances of 2.7 Å and 2.6 Å, as well as a minor increase in the bond distance with atoms comparable to the wild-type, were noticed (Fig 7).

### Molecular docking

**Fig 8:**
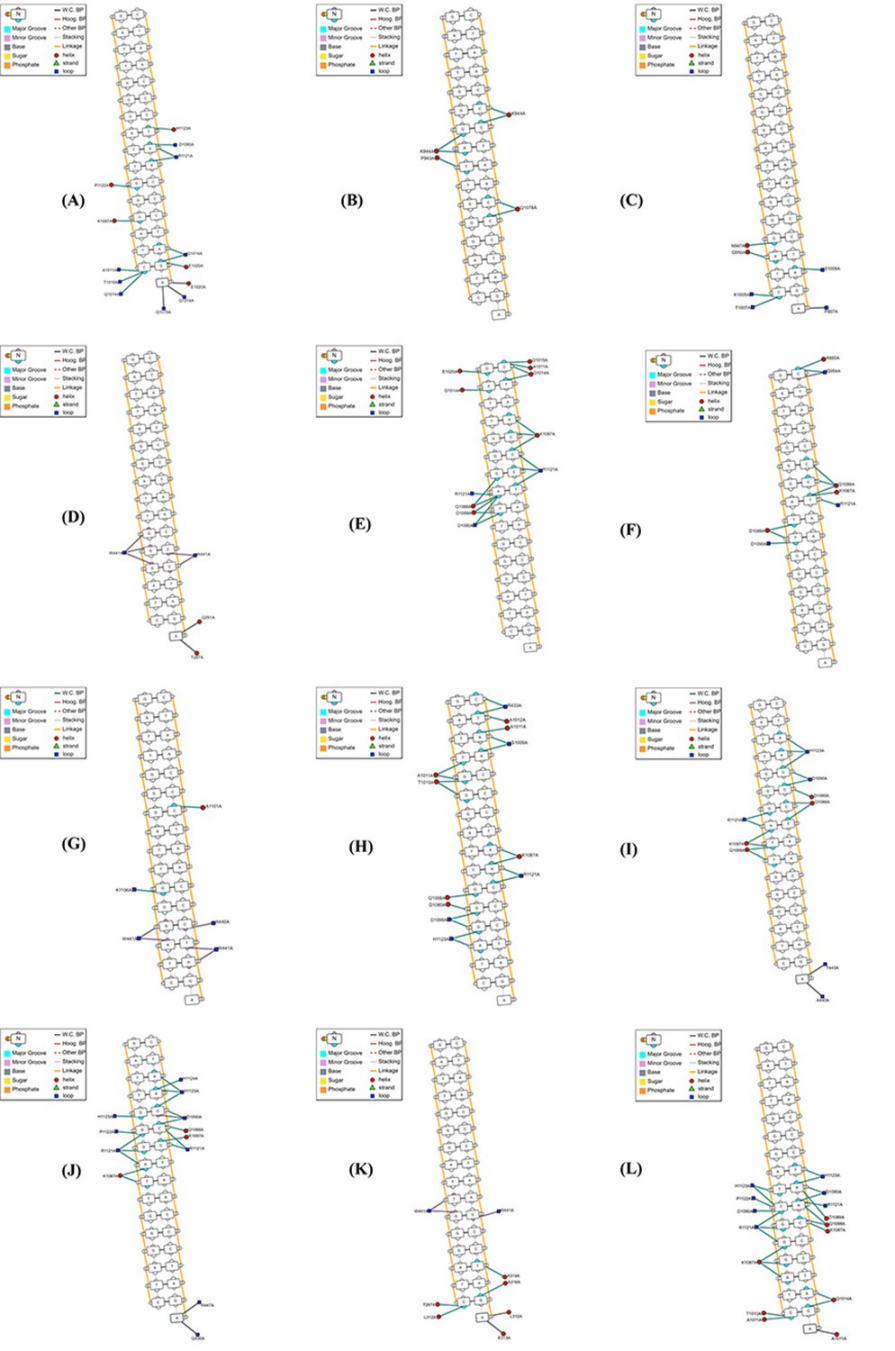
Graphical representation of molecular docking using DNAproDB. Illustration of docking result of DNA with (A) native and mutant (B) F15L, (C) M25V, (D)R141Q, (E) A252V, (F) G480R, (G) R639H, (H)G645D, (I) R697Q, (J) R700Q, (K) G706R, and (L) H960R protein are shown.

Due to RTEL1 being an essential DNA helicase, molecular docking of native and 11 filtered mutant proteins was performed with telomeric DNA (Fig 8). Active residues of the HHD2 domain in RTEL1 were extracted from the literature and used for specifying the DNA binding site in the HDOCK docking server.

A total of 12 molecular dockings were performed in the HDOCK server, which predicts binding complexes using a hybrid algorithm to predict binding affinity. Some deviation in the orientation of the molecular complexes has been observed. Six mutants (F15L, M25V, A252V, G480, R639H, and R697Q) have been predicted to have a less negative docking score when binding with DNA than the wild type, indicating a less stable binding complex. Besides, five of the other mutants showed a more negative docking score, which might result in a more rigid binding complex, leading to a discrepancy in the functionality of proteins (Table 7). The bound conformations revealed significant differences between the mutant and wild-type molecules when visualized in the DNAproDB web-based tool. All the mutant proteins deviated from the wild type when binding to DNA, not only in terms of interacting residues but also in the number of hydrogen bonds, Van der Waals interactions, and nucleic acid interactions. Additionally, the DNA has been observed to bind with entirely new residues in the F15L, M25V, and G706R mutant proteins compared to the wild type.

**Table 7:** Analysis of wild-type and mutant protein’s binding affinity and interaction with DNA.

Docked Molecules	Interacting Residues	Total HW^a^ Count	Total VdW^b^ count	Total Nucleotide Interaction Count	Docking Score	Confidence Score	Ligand RMSD (Å)
AF-DNA	H1123,D1090,R1121, P1122,K1087,Q1014, A1011,T1010, Q1015,E1020	9	70	25	-162.78	0.5636	163.85
F15L-DNA	P943,K944,Q1078	2	40	11	-108.52	0.3037	150.61
M25V - DNA	N947,P887,Q950, K1005,T1007,S1009	1	2	6	-147.45	0.4873	150.56
R141Q - DNA	R433,A1011,K1087, R1121,P1122,D1090, H1123,Q1126	1	45	19	-199.3	0.7283	159.89
A252V - DNA	A1011,E1020,K1087, Q1014,Q1015,Q1088, R1121	2	57	26	-137.35	0.4371	158.6
G480R - DNA	R895,Q954,Q1088, K1087,R1121, D1089,D1090	0	19	11	-129.01	0.3966	166.48
R639H - DNA	A440,A1101, K1106,W441	0	24	11	-137.36	0.4371	147.05
G645D - DNA	R433,S1009,T1010, A1011,A1012,K1087, Q1088,D1089,D1090, R1121,H1123	5	46	23	-180.79	0.6493	154.45
R697Q-DNA	A440,T443,K1087, Q1088,D1089,D1090, R1121, H1123	3	40	16	-147.01	0.4851	160.21
R700Q-DNA	Q436,R447,K1087, Q1088,D1090,R1121, P1122, H1123, H1124	5	72	20	-177.62	0.6347	156.1
G706R-DNA	E312,E313,T287, A316,K319,W441	0	21	9	-186.84	0.6763	146.61
H960R-DNA	T1010,A1011,Q1014, K1087, Q1088,D1089, D1090,R1121,P1122, H1123	7	82	24	-196.81	0.7183	166.68

^a^HW: H-bonding

^b^VdW: Van der Waals

### 5’ and 3’ UTR non-coding SNPs analysis

A total of 7 non-coding SNPs were extracted from the Ensemble database with the global minor allelic frequency (MAF) value ranging from 0.01 to 0.5. While analyzing the RegulomeDB database, rs1291208 scored 0.95 with rank 1a, indicating eQTL/caQTL, TF binding, matched TF motif, matched Footprint, and chromatin accessibility peak. Besides, rs114023340 scored 0.71269 and ranked 2a, indicating TF binding, matched TF motif, matched Footprint, and chromatin accessibility peak, and the rest of the SNPs (rs2297432, rs13043797, rs2297441, rs1291209, rs1295810) were scored 0.55436 and ranked 1f, which indicates eQTL/caQTL, TF binding/chromatin accessibility peak. Moreover, probability scores close to 1 indicates the likelihood of an SNP being a regulatory variant. Finally, the SNPs were analyzed in PolymiRTS Database 3.0, where out of seven SNPs, the CLASH system predicted only one (rs2297441) SNP in the target region of hsa-miR-615-3p.

## Discussion

As an essential DNA helicase, RTEL1 plays a vital role in the regulation and maintenance of telomeres. RTEL1 dissembles recombination intermediates, breaks down telomeric loops or T loops, and restricts excessive meiotic crossing over [6, 22]. Studies have shown the function of RTEL1 in DNA replication machinery and its association with maintaining the proper DNA replication, stability of replication fork, and maintenance of telomere integrity [22, 87]. In humans, mutations in the RTEL1 gene have been proven to cause a rare genetic hereditary disease called Dyskeratosis congenita (DC) and its severe form Hoyeraal–Hreidarsson syndrome (HHS). The deficiency of RTEL1 in different cell lines has proven the increasing risk of telomere fragility and genomic instability [2]. RTEL1 expression dysregulation or structural alteration may significantly contribute to the emergence of malignancies. Studies have shown that the RTEL1 genomic locus is often amplified in human cancers [21, 88, 89] and the polymorphisms of this gene are associated with several cancers, including gliomas, neuroblastoma, lung, and breast cancer [19, 20, 90, 91]. Additionally, it has also been found that genetic variations of the RTEL1 gene are linked to an elevated risk of stroke [92]. Though RTEL1 mutations and their association with human disorders are well-documented in studies, the full spectrum of polymorphic variations in RTEL1 and their effects on its biological functions remain largely unexplored. Therefore, in this study, we employ comprehensive *in silico* analysis to identify and characterize the most deleterious coding and non-coding SNPs in the RTEL1 gene and assess their impact on the structure and functionality of the protein.

Our initial classification of nsSNPs was based on how they might affect the structure and functionality of RTEL1 protein. Different bioinformatics tools have different threshold cut-off values for classifying SNPs as damaging or benign, which can occasionally lead to misleading predictions for SNPs with prediction scores close to the threshold cut-off value. Therefore, 19 web tools depending on the structural and sequential homology approaches were used to overcome this limitation to predict functionally and structurally deleterious nsSNPs. For the analysis, we employed the isoform 2 (1219 amino acid) sequence, as it is represented as a canonical sequence in the Uniport database. Using ten computational SNP prediction tools—SIFT, PROVEAN, Polyphen-2, PANTHER, SuSPect, PredictSNP, PredictSNP2, P-Mut, SNAP2, and SNP&GO—we screened out 43 significantly harmful nsSNPs from the 1392 nsSNPs mentioned in the NCBI dbSNP database. Based on the prediction scores produced by these ten web tools, the 43 harmful nsSNPs were chosen. The structural impact of the filtered nsSNPs was analyzed in two categories—mCSM, SDM, Duet, I-Mutant, INPS-MD, MuPro, and Dynamut2 was used for the prediction of stability change, whereas Mutpred2 and Project HOPE were utilized for phenotypic effects prediction.

**Fig 9:**
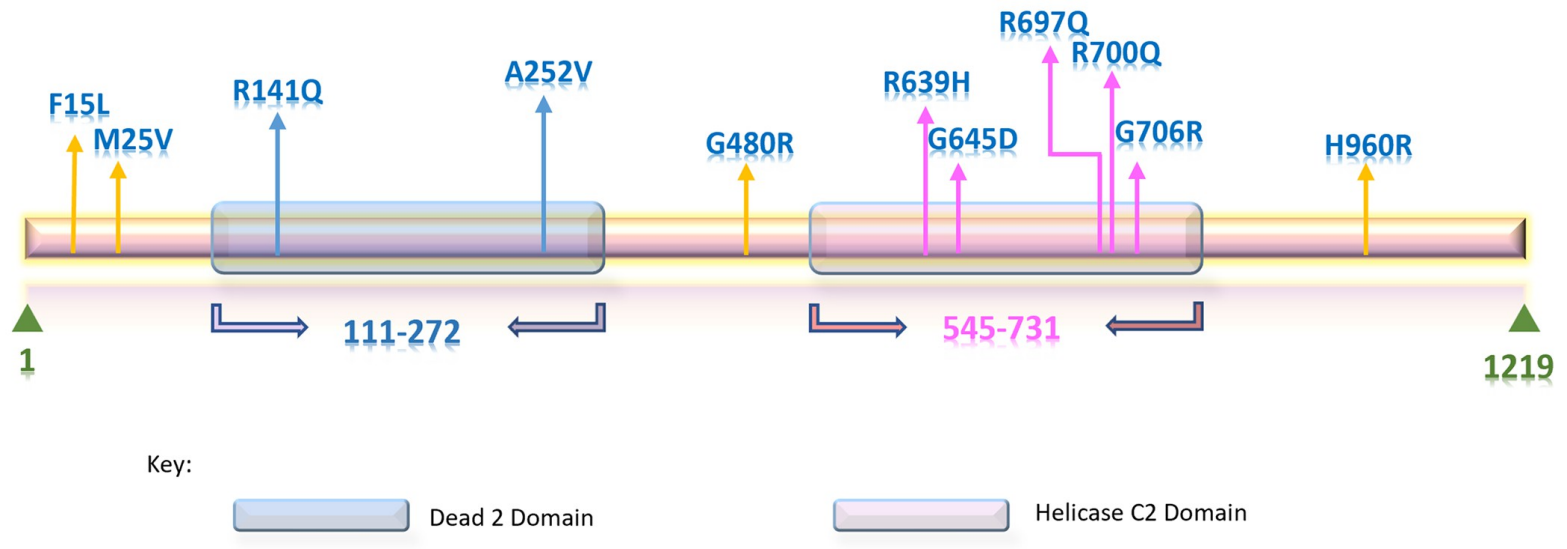
The final positions of 11 nsSNPs are shown within the RTEL1 protein. The domains are represented by transparent blue (Dead 2 domain) and light purple (Helicase C2 domain) horizontal bars, and nsSNPs are displayed as arrows where blue arrows indicate the nsSNP that falls under the Dead 2 domain, pink arrows depict that are in the Helicase C2 Domain and yellow indicates the one that does not belong to any domains. Both the scaling of the domains and nsSNPs positions are provided as approximations.

Protein stability, which governs protein conformational shape, determines how well a protein performs its function. Protein misfolding, disintegration, or aberrant protein aggregation can occur due to any alteration to the stability of the protein [93]. According to research, amino acid changes that reduce the stability of proteins by a few kcal/mol account for 80% of missense mutations linked to diseases [94]. The ΔΔG value we received as an output from the tools was used to assess the pathogenicity and the consequences of SNPs on the protein’s stability. The folding free energy change, or ΔΔG, separates the mutant from the wild type, which measures the effect of mutation on the protein’s stability [95]. Hence, a decline in ΔΔG value implies the mutant protein is losing its stability. Thus, we concentrated on the effects of the 43 harmful nsSNPs on the stability of the RTEL1 protein. Of these 43 nsSNPs, 13 nsSNPs (F15L, M25V, R141Q, A252V, G480R, F559L, R639H, G645D, R697Q, R700Q, G706R, R729C, H960R) were commonly predicted to have negative ΔΔG value by seven web servers, indicating a destabilizing effect on the protein. The phenotypic consequences of these variants were examined through MutPred2 and HOPE where MutPred2 predicted every potential gain, loss, or modification of different molecular properties, and HOPE thoroughly examined them. Except for the F559L and R729C mutations, all of the mutations were predicted to have a damaging effect on the protein (Fig 9). SNPs with glycine as wild-type residues (G480R, G645D, G706R) are highly conserved due to their small size and less steric hindrance of side chains, a crucial aspect for protein flexibility. Therefore, the flexibility required for protein function is compromised by its replacement [96]. Additionally, conformational flexibility is the primary factor influencing the aggregation tendency of protein. Thus, any alteration in protein flexibility may increase the likelihood of protein being aggregated and forming fibril [97, 98]. Moreover, arginine is a positively charged amino acid; variants where arginine is replaced with neutral or less basic amino acids (R141Q, R639H, R697Q, R700Q,) may lead to loss of interaction with other molecules, whereas in the case of H960R, it is predicted by HOPE to cause repulsion of ligand or other molecules of similar charges. Apart from these, changes in size and hydrophobicity due to the SNPs may also result in a destabilizing effect on proteins or a potential loss of external interactions. Because of the disparity in size, M25V is projected to result in a vacant space in the core of the protein. This result was also verified through the evaluation of interatomic interactions where all of the mutations have been observed to gain or lose some interactions with nearby atoms due to the substitution of amino acids. The most significant changes were observed in R141Q, G480R, R639H, G645D, R700Q, and G706R mutations. Besides, the domain and cluster information of these 11 nsSNPs were identified through Mutation3D. Two domains were identified in the RTEL1 protein where R141Q and A252V mutations are in the Dead 2 domain and R639H, G645D, R697Q, R700Q, and G706R, mutations are in the Helicase C2 domain. Also, 4 mutations (R639H, R697Q, R700Q, G706R) were found to form a cluster. Dead 2 domain is a part of RAD3-related DNA binding helicases involved in DNA repair, regulation of transcription, and metabolic process of nucleic acid and nucleotide. Whereas the Helicase C2 domain falls under the C terminal helicase domain, which is thought to be necessary for helicase activity [4] and the common phenotypic outcome seen in patients with HHS or DC, particularly short telomeres is predicted to be responsible for the altered activity of C terminal domain [4, 12]. Therefore, the mutations in these two domains of RTEL1 protein could impose a more deleterious effect.

Moreover, mutations in cancer tissues tend to form clusters in specific positions of protein [99]. It is worth mentioning here that evaluation of oncogenic susceptibility revealed the oncogenic potential of all of the 11 nsSNPs, and 2 (G480R, and G706R) of them were found to be directly associated with liver, and endometrial cancer. Thus, cluster-forming mutations could cause diseases due to the damaging impact on the protein’s functionality.

In secondary structure analysis, it has been found that all 11 mutations contain fewer beta and gamma turns than the wild type. All mutant structures displayed a larger RMSD value when mutant and wild-type structures were superimposed, which justifies the structural deviation resulting from single amino acid substitution in the protein. Although changing the seed value may slightly alter the structural configuration and hence the RMSD, we maintained consistency by using the same default seed value for generating all mutant structures. We acknowledge that the slight discrepancies in RMSD values could be influenced by the methods utilized in creating the structures. Our use of consistent modeling parameters was aimed at minimizing these discrepancies.

Additionally, evolutionary conservation of the protein sequence plays an essential role in evaluating the adverse effect of mutation on species. Therefore, using the ConSurf server, first, we identified the evolutionary conservation profile of each amino acid position in the RTEL1 protein, where all of the SNP positions were predicted to be conserved in the protein. For further evaluation, we executed multiple sequence alignments of ten species using MEGA11 software, and the result showed that all 11 positions are conserved in ten species. The phylogenetic tree also showed that the closest relatives of the human RTEL1 protein are orthologs in the primate species, chimpanzees, and gorillas.

The molecular docking analysis of telomeric DNA with native and 11 nsSNPs revealed alterations in binding affinity, which point to a shift in the interaction pattern of the complex. Usually, the better orientated the ligand is at the binding pocket of the receptor, the more negative the binding affinity becomes [100]. Hence, less negative binding affinity demonstrates the change in the binding orientation of the ligand to the receptor molecule, resulting from the substitution of amino acid residues. Out of 11 mutations, six mutations—F15L, M25V, A252V, G480, R639H, and R697Q were found to have less negative docking score than the wild-type protein, indicating a less stable binding complex. On the other hand, compared to the complex generated by the wild-type protein, mutations like R141Q, G645D, R700Q, G706R, and H960R revealed a stiffer DNA binding complex with a more negative docking score. Moreover, there was a discernible reduction of H-bond and Van der Waals interactions in the binding pocket.

Interestingly, a remarkable change in the receptor-interacting residues has been observed in F15L, M25V, and G706R mutations, where the DNA was found to bind with an entirely distinct set of residues than the wild-type. Additionally, nsSNPs F15L, R141Q and R697Q identified in our analysis were also reported in the ClinVar database. These variants were specifically associated with diseases such as dyskeratosis congenita, pulmonary fibrosis, bone marrow failure and telomere-related diseases. However, the clinical significance of these variants was categorized as uncertain. This ambiguity suggests that the available data is insufficient to confirm a definitive pathogenic role of these variants, despite some evidence linking them to certain disorders. On that point, our study provides definitive *in silico* evidence about the potential pathogenic role of these variants. Among the non-coding SNPs, two of them (rs1291208 & rs114023340) were predicted to have the most likelihood of having a regulatory influence on RTEL1 protein as the exhibited predictions involved eQTL/caQTL, TF binding, matched TF motif, matched Footprint, and chromatin accessibility. Furthermore, rs2297441 was predicted in a miRNA’s target region, and its presence may impede the regulation of RTEL1 by miR-615-3p. miR-615-3p plays a multifaceted role in cancer, promoting proliferation, migration, and inhibiting apoptosis in gastric cancer, enhancing adverse outcomes in prostate cancer, facilitating the epithelial-mesenchymal transition and metastasis in breast cancer, and participating in the repression of hTERT and tumorigenesis in collaboration with HoxC5, while also promoting hypoxia-induced glycolysis in non-small cell lung cancer through interaction with HMGB3 [101–104]. It is noteworthy that the majority of the single-nucleotide polymorphisms/variants (SNPs/ SNVs) that have been discovered through genome-wide association studies (GWAS) as risk factors for complex diseases, commonly reside within non-coding regions of the genome [105–112]. Therefore, the presence of SNPs within the non-coding region can have a substantial impact on the regulatory elements and pathways involved in disease susceptibility progression.

The findings reported in this study have several important implications for clinical practice and research. The identified deleterious variants could be integrated into genetic diagnostic panels, improving the accuracy of risk assessments for patients with RTEL1-related disorders. This would facilitate earlier and more precise diagnosis of conditions like DC and HHS, potentially leading to timely interventions and personalized management strategies. Understanding the specific mutations that affect RTEL1 function can pave the way for personalized therapeutic approaches. For instance, mutations like F15L, M25V, A252V, G480, R639H, and R697Q which significantly alter DNA binding affinity, could be potential targets for drug development to compensate for potential functional losses. Future research should focus on experimentally validating these *in silico* findings through laboratory techniques and cellular models to confirm the functional impacts of the identified variants. Additionally, to further confirm the oncogenic potential of the nsSNPs identified in our study, high-risk variants such as G480R and G706R should be examined in the context of liver and endometrial cancers, while the other highly oncogenic variants (R639H, G645D, R697Q, R700Q) also warrant detailed exploration. Understanding how these mutations contribute to cancer development and progression could provide valuable insights into their potential as biomarkers for cancer susceptibility and their role in the malignancy process.

## Conclusion

Our study identified 11 nsSNPs and 3 non-coding SNPs of the RTEL1 gene that are predicted to be deleterious. These mutations were discovered to have a deleterious impact on the structural and functional properties of the RTEL1 protein, which may disrupt the conformation of the native protein. This extensive study can, therefore, be constructive in future research on RTEL1, opening the door to the possibility of looking into potential disease-causing SNPs and facilitating the identification of potent drugs or pharmacological targets. Hence, experimental mutational research, genome-wide association studies, and clinical-based studies are further required to validate these findings.

## Supporting information

S1 Fig The 11 mutated structures aligned with the wild type structure. (TIF)

S1 Table Prediction of functionally damaging nsSNPs determined by 10 bioinformatics web tools with dbSNP ID. (DOCX)
