# Supplementary figures and images for: A comprehensive in silico investigation into the pathogenic SNPs in the RTEL1 gene and their biological consequences
Source: PLoS One. 2024 Sep 6;19(9):e0309713. doi: 10.1371/journal.pone.0309713 (PMC11379182; doi:10.1371/journal.pone.0309713)

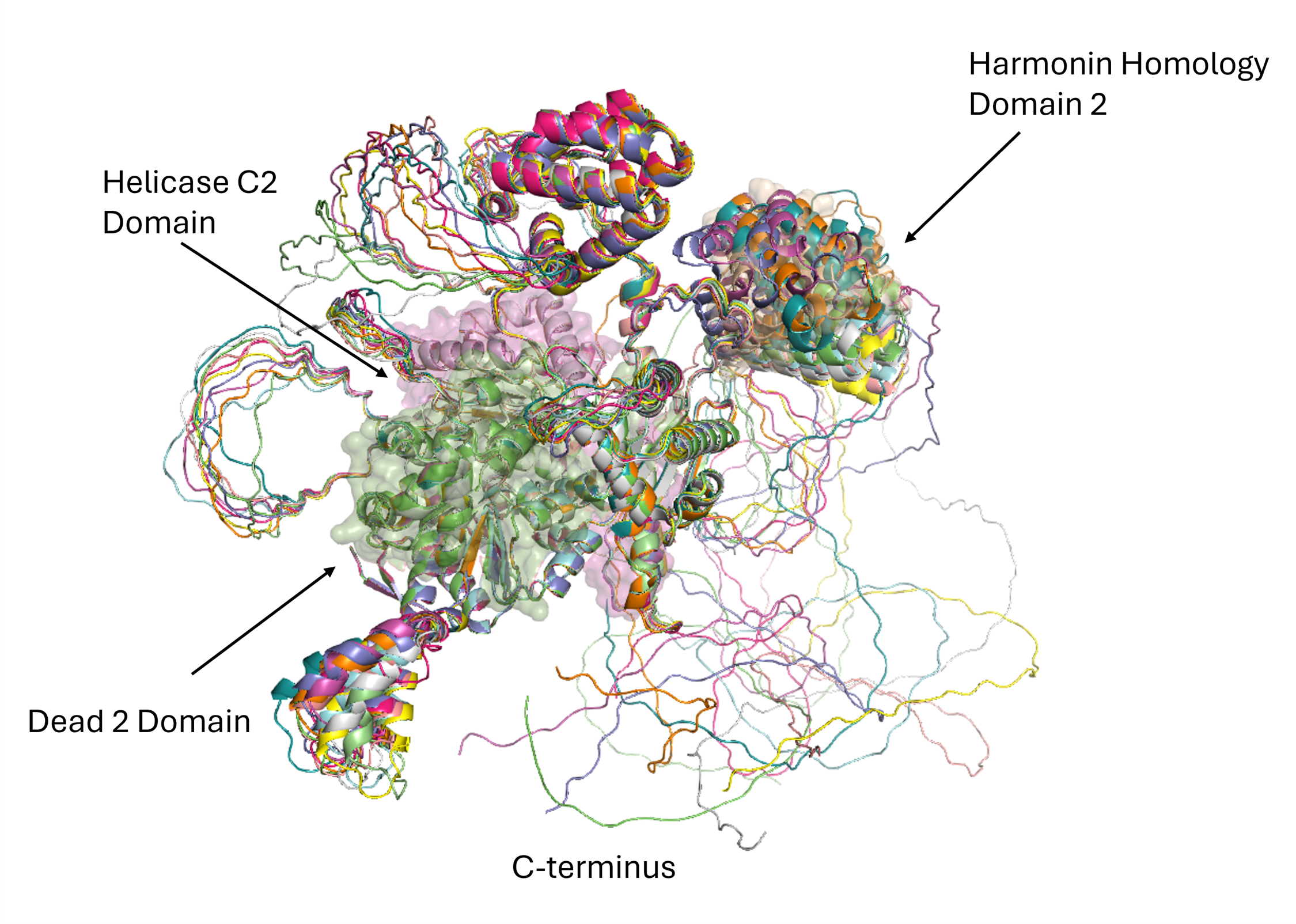

Supplement: S1 Fig — (TIF) [file pone.0309713.s001.tif]
