# Supplementary material for: A comprehensive in silico investigation into the pathogenic SNPs in the RTEL1 gene and their biological consequences
Source: PLoS One. 2024 Sep 6;19(9):e0309713. doi: 10.1371/journal.pone.0309713 (PMC11379182; doi:10.1371/journal.pone.0309713)
Supplement: S1 Table — (DOCX) [file pone.0309713.s002.docx]

**S1 Table****. Prediction of functionally damaging nsSNPs determined by 10 bioinformatics web tools with dbSNP ID.**

| **Serial No.** | **SNP ID** | **Variants** | **SIFT** | **PROVEAN** | **PolyPhen2** | **Panther** | **SuSPect** | **PredictSNP** | **PredictSNP2** | **P-Mut** | **SNAP2** | **SNP &GO** |
| --- | --- | --- | --- | --- | --- | --- | --- | --- | --- | --- | --- | --- |
| 1 | rs1296968885 | F15L | Deleterious | Deleterious | Damaging | Damaging | Disease | Deleterious | Deleterious | Disease | Effect | Disease |
| 2 | rs2089974057 | M25V | Deleterious | Deleterious | Damaging | Damaging | Disease | Deleterious | Deleterious | Disease | Effect | Disease |
| 3 | rs1044603913 | T44M | Deleterious | Deleterious | Damaging | Damaging | Disease | Deleterious | Deleterious | Disease | Effect | Disease |
| 4 | rs1163455875 | T49M | Deleterious | Deleterious | Damaging | Damaging | Disease | Deleterious | Deleterious | Disease | Effect | Disease |
| 5 | rs1407364310 | L50P | Deleterious | Deleterious | Damaging | Damaging | Disease | Deleterious | Deleterious | Disease | Effect | Disease |
| 6 | rs778739638 | C54F | Deleterious | Deleterious | Damaging | Damaging | Disease | Deleterious | Deleterious | Disease | Effect | Disease |
| 7 | rs554402067 | S113P | Deleterious | Deleterious | Damaging | Damaging | Disease | Deleterious | Deleterious | Disease | Effect | Disease |
| 8 | rs963067934 | T115S | Deleterious | Deleterious | Damaging | Damaging | Disease | Deleterious | Deleterious | Disease | Effect | Disease |
| 9 | rs746010778 | H116P | Deleterious | Deleterious | Damaging | Damaging | Disease | Deleterious | Deleterious | Disease | Effect | Disease |
| 10 | rs1333433189 | S140F | Deleterious | Deleterious | Damaging | Damaging | Disease | Deleterious | Deleterious | Disease | Effect | Disease |
| 11 | rs746931551 | R141W | Deleterious | Deleterious | Damaging | Damaging | Disease | Deleterious | Deleterious | Disease | Effect | Disease |
| 12 | rs1242481082 | R141Q | Deleterious | Deleterious | Damaging | Damaging | Disease | Deleterious | Deleterious | Disease | Effect | Disease |
| 13 | rs772748212 | P225L | Deleterious | Deleterious | Damaging | Damaging | Disease | Deleterious | Deleterious | Disease | Effect | Disease |
| 14 | rs1188469323 | N227D | Deleterious | Deleterious | Damaging | Damaging | Disease | Deleterious | Deleterious | Disease | Effect | Disease |
| 15 | rs923910999 | Y228C | Deleterious | Deleterious | Damaging | Damaging | Disease | Deleterious | Deleterious | Disease | Effect | Disease |
| 16 | rs764165415 | D231V | Deleterious | Deleterious | Damaging | Damaging | Disease | Deleterious | Deleterious | Disease | Effect | Disease |
| 17 | rs398123019 | E251K | Deleterious | Deleterious | Damaging | Damaging | Disease | Deleterious | Deleterious | Disease | Effect | Disease |
| 18 | rs1454150484 | A252V | Deleterious | Deleterious | Damaging | Damaging | Disease | Deleterious | Deleterious | Disease | Effect | Disease |
| 19 | rs748740521 | H253Y | Deleterious | Deleterious | Damaging | Damaging | Disease | Deleterious | Deleterious | Disease | Effect | Disease |
| 20 | rs1386490624 | P460L | Deleterious | Deleterious | Damaging | Damaging | Disease | Deleterious | Deleterious | Disease | Effect | Disease |
| 21 | rs2090629066 | T478N | Deleterious | Deleterious | Damaging | Damaging | Disease | Deleterious | Deleterious | Disease | Effect | Disease |
| 22 | rs1012871786 | G480R | Deleterious | Deleterious | Damaging | Damaging | Disease | Deleterious | Deleterious | Disease | Effect | Disease |
| 23 | rs773057452 | T481M | Deleterious | Deleterious | Damaging | Damaging | Disease | Deleterious | Deleterious | Disease | Effect | Disease |
| 24 | rs786205700 | P484L | Deleterious | Deleterious | Damaging | Damaging | Disease | Deleterious | Deleterious | Disease | Effect | Disease |
| 25 | rs374168761 | F559L | Deleterious | Deleterious | Damaging | Damaging | Disease | Deleterious | Deleterious | Disease | Effect | Disease |
| 26 | rs535749230 | A621V | Deleterious | Deleterious | Damaging | Damaging | Disease | Deleterious | Deleterious | Disease | Effect | Disease |
| 27 | rs753779060 | R624Q | Deleterious | Deleterious | Damaging | Damaging | Disease | Deleterious | Deleterious | Disease | Effect | Disease |
| 28 | rs2090674658 | S628G | Deleterious | Deleterious | Damaging | Damaging | Disease | Deleterious | Deleterious | Disease | Effect | Disease |
| 29 | rs766112578 | E629K | Deleterious | Deleterious | Damaging | Damaging | Disease | Deleterious | Deleterious | Disease | Effect | Disease |
| 30 | rs1262691904 | R639C | Deleterious | Deleterious | Damaging | Damaging | Disease | Deleterious | Deleterious | Disease | Effect | Disease |
| 31 | rs1484865003 | R639H | Deleterious | Deleterious | Damaging | Damaging | Disease | Deleterious | Deleterious | Disease | Effect | Disease |
| 32 | rs1262230406 | G640D | Deleterious | Deleterious | Damaging | Damaging | Disease | Deleterious | Deleterious | Disease | Effect | Disease |
| 33 | rs367688683 | G645D | Deleterious | Deleterious | Damaging | Damaging | Disease | Deleterious | Deleterious | Disease | Effect | Disease |
| 34 | rs1323023332 | L646F | Deleterious | Deleterious | Damaging | Damaging | Disease | Deleterious | Deleterious | Disease | Effect | Disease |
| 35 | rs1177091623 | P647L | Deleterious | Deleterious | Damaging | Damaging | Disease | Deleterious | Deleterious | Disease | Effect | Disease |
| 36 | rs16983886 | K659N | Deleterious | Deleterious | Damaging | Damaging | Disease | Deleterious | Deleterious | Disease | Effect | Disease |
| 37 | rs760108811 | G696R | Deleterious | Deleterious | Damaging | Damaging | Disease | Deleterious | Deleterious | Disease | Effect | Disease |
| 38 | rs1176364985 | R697Q | Deleterious | Deleterious | Damaging | Damaging | Disease | Deleterious | Deleterious | Disease | Effect | Disease |
| 39 | rs1416515129 | R700Q | Deleterious | Deleterious | Damaging | Damaging | Disease | Deleterious | Deleterious | Disease | Effect | Disease |
| 40 | rs1332347156 | G706R | Deleterious | Deleterious | Damaging | Damaging | Disease | Deleterious | Deleterious | Disease | Effect | Disease |
| 41 | rs1472657490 | P725R | Deleterious | Deleterious | Damaging | Damaging | Disease | Deleterious | Deleterious | Disease | Effect | Disease |
| 42 | rs1555811919 | R729C | Deleterious | Deleterious | Damaging | Damaging | Disease | Deleterious | Deleterious | Disease | Effect | Disease |
| 43 | rs2090803986 | H960R | Deleterious | Deleterious | Damaging | Damaging | Disease | Deleterious | Deleterious | Disease | Effect | Disease |
